# Deciphering mechanisms underlying the genetic variation of general production and liver quality traits in the overfed mule duck by pQTL analyses

**DOI:** 10.1186/s12711-017-0313-6

**Published:** 2017-04-19

**Authors:** Yoannah François, Alain Vignal, Caroline Molette, Nathalie Marty-Gasset, Stéphane Davail, Laurence Liaubet, Christel Marie-Etancelin

**Affiliations:** 1 GenPhySE, INRA, INPT, INP-ENVT, Université de Toulouse, 31326 Castanet Tolosan, France; 2 IPREM-EMM, UMR5254, Université de Pau et des Pays de l’Adour, 40004 Mont de Marsan Cedex, France

## Abstract

**Background:**

The aim of this study was to analyse the mechanisms that underlie phenotypic quantitative trait loci (QTL) in overfed mule ducks by identifying co-localized proteomic QTL (pQTL). The QTL design consisted of three families of common ducks that were progeny-tested by using 294 male mule ducks. This population of common ducks was genotyped using a genetic map that included 334 genetic markers located across 28 APL chromosomes (APL for *Anas platyrhynchos*). Mule ducks were phenotyped for 49 traits related to growth, metabolism, overfeeding ability and meat and fatty liver quality, and 326 soluble fatty liver proteins were quantified.

**Results:**

One hundred and seventy-six pQTL and 80 phenotypic QTL were detected at the 5% chromosome-wide significance threshold. The great majority of the identified pQTL were trans-acting and localized on a chromosome other than that carrying the coding gene. The most significant pQTL (1% genome-wide significance) were found for alpha-enolase on APL18 and fatty acid synthase on APL24. Some proteins were associated with numerous pQTL (for example, 17 and 14 pQTL were detected for alpha-enolase and apolipoprotein A1, respectively) and pQTL hotspots were observed on some chromosomes (APL18, 24, 25 and 29). We detected 66 co-localized phenotypic QTL and pQTL for which the significance of the two-trait QTL (2t-QTL) analysis was higher than that of the strongest QTL using a single-trait approach. Among these, 16 2t-QTL were pleiotropic. For example, on APL15, melting rate and abundance of two alpha-enolase spots appeared to be impacted by a single locus that is involved in the glycolytic process. On APLZ, we identified a pleiotropic QTL that modified both the blood level of glucose at the beginning of the force-feeding period and the concentration of glutamate dehydrogenase, which, in humans, is involved in increased glucose absorption by the liver when the *glutamate dehydrogenase 1* gene is mutated.

**Conclusions:**

We identified pleiotropic loci that affect metabolic pathways linked to glycolysis or lipogenesis, and in the end to fatty liver quality. Further investigation, via transcriptomics and metabolomics approaches, is required to confirm the biomarkers that were found to impact the genetic variability of these phenotypic traits.

**Electronic supplementary material:**

The online version of this article (doi:10.1186/s12711-017-0313-6) contains supplementary material, which is available to authorized users.

## Background

To date, approaches based on transcript abundance quantitative trait loci (QTL), better known as expression QTL (eQTL) have been the primary method used to understand the genetic architecture that underlies physiological traits controlled by QTL and the relationships between the genome and the phenome. However, the measure of transcript abundance used for eQTL analysis does not necessarily reflect the real abundance of the proteins coded by the genes. Indeed, mRNA levels can be influenced by multiple and complex regulation processes, which, for instance, affect transcription levels or mRNA stability, whereas protein abundance depends also on other levels of regulation, such as translation, maturation, post-translational modification or protein degradation. Proteomic analyses can be performed to determine whether the protein is an inactive propeptide or in a modified active state. A study by Darmeval et al. [1] showed that there is a link between protein abundance and genome variability, which suggests that quantitative proteomic analyses are a better indicator of genetic distances between maize lines than qualitative analyses. These authors later introduced the concept of PQL (protein quantitative loci), hereafter designated pQTL for consistency with the current nomenclature of eQTL, when they successfully mapped loci that influence protein abundance [2]. One key benefit of the identification of eQTL and pQTL lies in their possible co-location with phenotypic QTL, thus highlighting the importance of specific proteins or candidate genes, as was shown in a study on pea [3]. In the animal kingdom, research on pQTL is more recent, but has proven effective in finding genes that cause variation in plasmatic protein abundance in mice, and one of these genes was linked to both a pQTL and a QTL for HDL cholesterol levels [4]. To our knowledge, no pQTL analyses have been performed on animals of agricultural interest. Although various large-scale studies on eQTL have already been implemented owing to the availability of adequate techniques, they need to be complemented by pQTL analyses, which are effective for a deeper understanding of phenotypes. Indeed, since proteins are the actual cellular effectors of many physiological processes, identification of the loci that control their availability and abundance is an essential step in understanding the links between the genome and the phenome.

In a previous study, we identified QTL that are related to force-feeding traits [5] and are of great interest to the duck industry. In the current study, we quantified the proteins that are present in the fatty liver of the same ducks by quantitative 2D-gel electrophoresis [6] in order to perform pQTL analyses. The co-localized QTL and pQTL were investigated in an attempt to make connections between the phenotype and the proteome, and thus identify the biological mechanisms that underlie the genetic variability of these traits. Here, we present the results of QTL analyses on fatty liver proteomic data by focusing on the proteomic and phenotypic QTL that co-localized, and identified those that appear to have pleiotropic effects.

## Methods

Experimental procedures were performed in accordance with the French National Guidelines for the care and use of animals for research purposes (Certificate of Authorization to Experiment on Living Animals no 7740, Ministry of Agriculture and Fish Products).

### Experimental design and animal husbandry

**Fig. 1:**
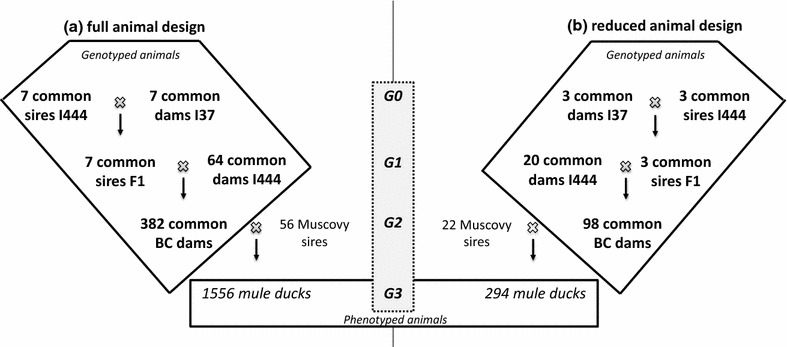
Experimental designs. I444: INRA Kaiya line; I37: INRA heavy Pekin line; BC: backcross

Due to the complexity of generating the proteomic dataset on a large scale, the experimental design used here (Fig. 1b) is a subset of the complete design (Fig. 1a) used by Kileh-Wais et al. [5]. Briefly, it consists of a backcross (BC) design in which an additional generation (G) of overfed male mule ducks (G3) was phenotyped to estimate the value of their G2 common duck mothers. G0 animals were recruited in two experimental common duck lines: I444, a light Kaiya line (the crossbred product of a Tsaiya duck and an Asian Pekin duck) and I37, a heavy Pekin line (a synthetic strain created from three heavy European Pekin lines) [5]. The design was reduced by (1) selecting three F1 families from the initial seven, in which QTL for fatty liver quality traits segregated and (2) reducing the number of mule duck offspring per BC female down to three.

Breeding of the G3 mule ducklings is described in [5] and [7]. The 294 mule ducks of the subset used here, were hatched in two batches, with a 3-week gap between hatches. From 0 to 12 weeks of age, they were bred in growing batches and then were overfed for 12 days by three different handlers. At the end of the overfeeding period, animals were slaughtered, and liver tissue was sampled 20 min *post*-*mortem* via a small slit in the abdomen and frozen in liquid nitrogen for proteomics analyses. Carcasses were refrigerated for 24 h at 4 °C, prior to evisceration.

### Proteomic 2D electrophoresis and identification of spots

**Fig. 2:**
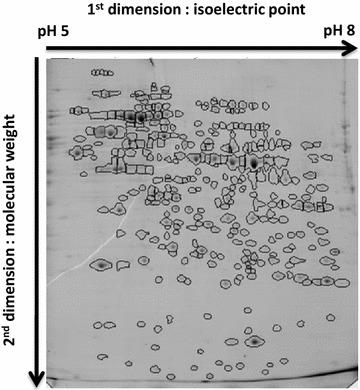
Two-dimensional gel electrophoresis representing a map of duck fatty liver soluble proteins

Bi-dimensional gel electrophoreses of protein extracts (Fig. 2) were performed for all 294 mule duck livers as reported by François et al. [6], according to the method described in [8]. Briefly, soluble protein fractions were extracted by grounding the frozen liver samples in liquid nitrogen, mixing them with a low ionic strength buffer, centrifuging the homogenates and collecting the supernatants. Protein concentrations were determined using the Bradford assay (Bio-Rad, Hercules, USA). For the first-dimensional electrophoresis, samples were loaded onto pH gradient strips (pH 5–8; Bio-Rad) and isoelectric focusing (IEF) was performed using a Protean IEF cell system (Bio Rad, Hercules, USA). The second dimension consisted of sodium dodecyl sulfate–polyacrylamide gel electrophoresis (SDS-PAGE) using a Protean II XL system (Bio Rad). IEF were processed in 30 series of 12 samples, and for each IEF series, SDS-PAGE were done in two series of six samples. SDS-PAGE gels were stained overnight with Coomassie Blue G250 (Fermentas Page Blue), scanned and analyzed with the Progenesis SameSpots software^®^ (TotalLab Ltd, Newcastle-upon-Tyne, UK). When spots seemed to be affected by the background, their outer edges were manually defined. As the general aspect of a gel had an impact on image analysis, gels were assigned to three categories: broken, blurred or correct. Spot matching was performed for all 294 samples and the software calculated the intensity that was corrected for background, of all of the spots detected for each of the 294 samples.

Detected spots were manually excised from the gels and sent to the proteomics platform in Clermont-Ferrand for protein identification (PFEMcp, INRA, Clermont-Ferrand Theix, France). In short, after protein digestion, peptide mixtures were analyzed by online nanoflow liquid chromatography using an Ultimate 3000 RSLC system (Dionex, Voisins le Bretonneux, France). Raw data were processed with Proteome Discoverer 1.4 (Thermo Fisher Scientific Inc., USA) and database searching with MASCOT v. 2.3 (Matrix Science Ltd., USA), using the UniP_tax_Aves database for protein identification. The genes that coded for the identified proteins were mapped on the chicken genome with Ensembl (http://www.ensembl.org). Since considerable synteny has been demonstrated between duck and chicken genomes, except for GGA4 (GGA for *Gallus gallus* chromosome), which is separated into two chromosomes in ducks, i.e. APL4 and 10 (APL for *Anas platyrhynchos* chromosome) [9], we considered that duck chromosomes APL1 to APL9 correspond to chicken chromosomes GGA1 to GGA9, APL10 corresponds to GGA4p and finally, that the rest of the karyotype is offset by one, with GGA10 corresponding to APL11 and so on. The list of all identified protein spots with pQTL is in Additional file 1: Table S1.

### Phenotypic data

Six groups of phenotypes corresponding to 49 traits were measured and recorded for the 294 mule ducks (Table 1). For growth traits measured before the overfeeding period, animal body weights were recorded at 12, 28, 42, and 70 days of age and the combinations of six body weight gains between these ages were estimated. Corticosterone levels under stress were recorded at 6 weeks of age: ducks were hung by the legs on a string for 10 min in order to measure the animal’s response to stress and blood samples were taken before and after the test in order to measure corticosterone levels and to assess the response of the HPA (hypothalamic–pituitary–adrenal) axis to this stress. Differences in corticosterone levels before and after stress were computed. During the overfeeding period, plasma metabolic indicators such as glucose, triglyceride and cholesterol levels were measured at the beginning (after the second meal), the middle (after the 10th meal) and the end (after the 20th meal) of the 12-day overfeeding period. Body weight at the beginning and the end of the overfeeding period, the corresponding body weight gain and the food consumption during the whole overfeeding period were recorded. To appreciate the overfeeding ability of the ducks, the carcass and component pieces (fatty liver, thigh, breast skin, breast muscle and abdominal fat) were dissected and weighed. Measurements related to liver quality such as melting rate (percentage of fat loss during cooking, obtained by sterilizing 60 g of liver for 50 min at 105 °C), lipid, protein and collagen contents, and liver color (L*, a*, b* coordinates in the CIELAB system) were recorded. Finally, breast muscle quality (*pectoralis major* muscle) was estimated by measuring the pH 20 min and 24 h (ultimate pH) *post*-*mortem*, cooking and drip losses under vacuum, the descriptive color L*, a*, b* values and by recording the lipid content. Raw meat tenderness was measured using the maximal shear force and energy levels using the Warner–Bratzler test. Mean and standard error values for all these traits are described in [5] and estimated genetic parameters are in [7].

**Table 1:** Trait descriptions

Abbreviation	Unit	Meaning
*Growth measurements*		
BW12, BW28, BW42, BW70	kg	Body weights at 12, 28, 42, 70 days of age
BWG12-28, BWG12-42, BWG12-70, BWG28-42, BWG28-70, BWG42-70	g/d	Body weight gains (all combinations between 12, 28, 42 and 70 days of age)
*Corticosterone traits*		
CortL, CortH	ng/ml	Corticosterone level before and after stress
DeltaC	ng/ml	Difference in corticosterone level before and after stress
*Body weights and metabolic traits during overfeeding period*		
TG 2nd M, TG 10th M, TG 20th M	g/l	Plasma triglyceride level after 2nd, 10th and 20th meal
CHO 2nd M, CHO 10th M, CHO 20th M	g/l	Plasma cholesterol level after 2nd, 10th and 20th meal
GLU 2nd M, GLU 10th M, GLU 20th M	g/l	Plasma glucose level after 2nd, 10th and 20th meal
DFI	kg/d	Daily feed intake
BWbeg, BWend	kg	Body weight at beginning and end of overfeeding period
OWG	kg	Weight gain during the overfeeding period
*Overfeeding ability traits*		
CW	kg	Bled-plucked carcass weight
FLW	kg	Fatty liver weight
pmMW	kg	*Pectoralis major* muscle weight
pmSFW	kg	Breast skin + subcutaneous fat weight
TSW	kg	Thigh + shank weight
AFW	kg	Abdominal fat weight
*Liver quality traits*		
MR	%	Liver melting rate
LLipC, LProtC	%	Liver lipid and protein content
LColC	mg/g	Liver collagen content
LL*, La*, Lb*		Liver lightness, redness and yellowness
*Muscle quality traits*		
MpH20, MpHu		Muscle pH 20 min *post mortem* and muscle ultimate pH 24 h *post mortem*
MCookL, MvacL	%	Muscle cooking losses and muscle drip losses
MLipC	%	Muscle lipid contents
ML*, Ma*, Mb*		Muscle lightness, redness and yellowness
Menergy	mJ	Energy needed to cut the muscle
MFmax		Maximal shear force

### Marker development, genotyping and map construction

The same BC design that was used here was previously used to detect phenotypic QTL based on a first set of 91 microsatellite markers, which led to the construction of 16 linkage groups that covered 778 cM [5]. In order to extend this rudimentary map, we developed additional single nucleotide polymorphisms (SNPs) [10]. Briefly, the seven G1 sires of the QTL design (Fig. 1a) were sequenced with 100 bp paired-end reads at a depth of 35X with the Illumina HiSeq. Sequence quality was verified and correct paired-end alignments were generated by alignment to the duck genome reference [11] using the Burrows-Wheeler Aligner (BWA) program [12], then SNPs were detected using the GATK software [13]. Over 11 million SNPs were detected, of which 90% were heterozygous in only one G1 sire. To guide our choice of SNPs, while allowing for the largest possible duck genome coverage, we took advantage of the known synteny conservation between the duck and chicken genomes [9, 14] and chose a final set of 384 SNPs among the 157,436 that were bi-allelic in at least five sires and had known positions on the chicken genome. These SNPs were used to genotype the 382 G2 female ducks, their G1 parents (seven F1 sires and 64 I444 dams) and their 14 G0 paternal grand-parents (Fig. 1a) using the Illumina^®^ Veracode technology. Analysis of genotype clusters and selection of high-quality SNPs, based on call rates and correct Mendelian inheritance, were performed with the Genome Studio™ software (Illumina).

**Fig. 3:**
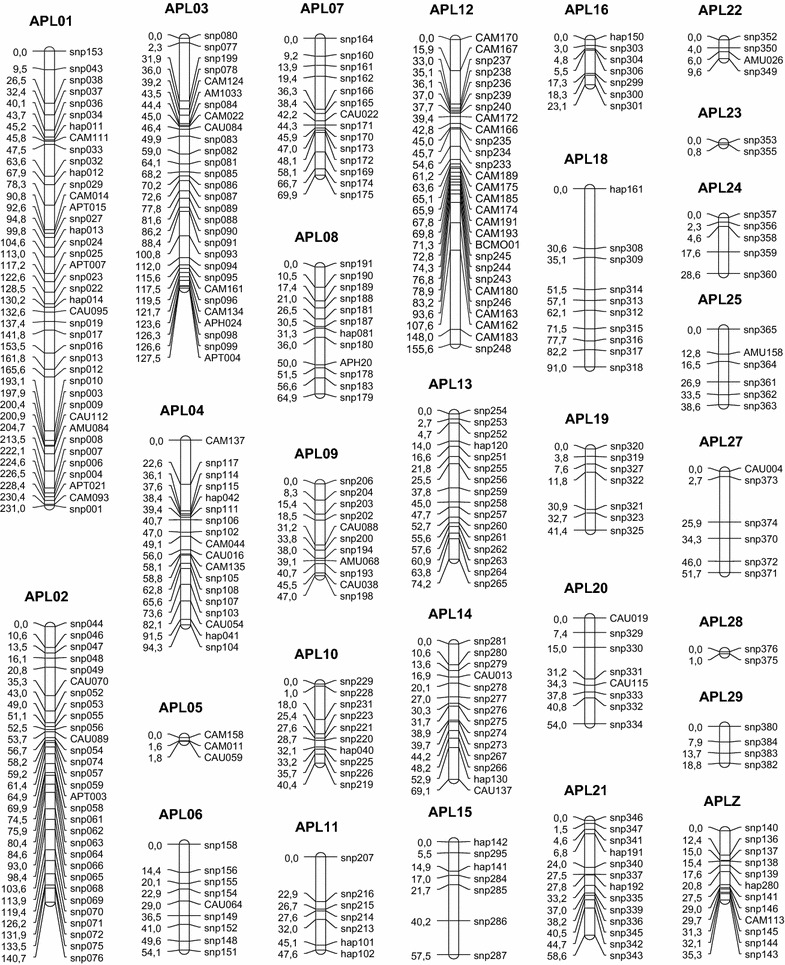
Sex-averaged genetic map in centiMorgan. Linkage groups (APL) were built using the Crimap software

Genetic maps were constructed with CRI-MAP 2.4 [15] by including the SNP genotypes generated here and previous microsatellite data. The new genetic map contains 334 markers (278 SNPs and 56 microsatellites) aggregated into 28 linkage groups corresponding to 28 APL chromosomes (Fig. 3).

### Statistical methods

Prior to QTL detection, all mule duck traits (proteomic or phenotypic) were corrected for environmental fixed effects using the GLM procedure in SAS [16]. For all phenotypic traits, the “hatching batch” effect (two levels) was taken into account (Model 1), and the “handler” effect (three levels) was added for traits related to overfeeding or product quality (Model 2). For proteomic traits, these zootechnical effects were cumulated with the technological effects of the bi-dimensional electrophoresis. Then, the sum of the spot intensities of each gel was treated as a covariate and six fixed effects were defined (Model 3): the “handler” effect (three levels), the “hatching batch” effect (two levels), the general aspect of the gels (three levels), the first electrophoresis dimension effect (30 levels), the second electrophoresis dimension effect (two levels per first electrophoresis series) and the interaction of both dimensions (60 levels). The residual effects of the three previous linear models were conserved for each mule duck, and the performance of each G2 female was computed as the average of the residual effects of her three male mule duck offspring.

QTL detection was carried out using the QTLMap software [17–19] in order to implement linkage analysis according to the interval mapping method [20]. For each chromosome, first the probabilities of each possible phase of the G1 male founders were estimated using marker information from their progenies (the G2 dams). The sire phases with the highest probabilities were assumed to be the correct ones: for a set of tested positions (practically at each 1 cM), the probabilities that the corresponding chromosomal segments were transmitted to the offspring were estimated. Then, QTL detection was carried out by within-sire linear regression [21]. The model was the following:

$$Y_{ij} = s_{i} + \left( {2p_{ij} - 1} \right)a_{i} + e_{ij} ,$$

where the dependent variable $Y_{ij}$ is the average performance (previously corrected for fixed effects) of the three male mule duck offspring of G2 dam $j$ and sire $i$. For each location on the genome, $s_{i}$ is the male founder $i$ effect, $a_{i}$ is equal to half the substitution effect of the putative QTL carried by the sire $i$, and $p_{ij}$ is the probability that the daughter (BC) $j$ might inherit one arbitrarily defined QTL allele from her sire $i$, given the marker information. The residual variance $e_{ij}$ was defined within sire families to improve robustness to unlinked QTL segregation between families [22]. In our design, phenotypes were recorded only at G3, but since the number of mule ducks per G2 dam was strictly equal to 3, it was not necessary to take the variance of the phenotypes assigned to the G2 generation into account, in contrast with our previous study in which the number of G3 mule ducks per G2 dam was variable [5].

For each trait and each linkage group, 1000 within-family permutations were performed to estimate the empirical chromosome-wide significance level of the test statistics [23]. The conservative genome-wide thresholds were derived from chromosome-wide significance levels, using an approximate Bonferroni correction:

$$P_{genome{\text -}wide} = 1 - \left( {1 - P_{chromosome{\text -}wide} } \right)^{{{\raise0.7ex\hbox{$1$} \!\mathord{\left/ {\vphantom {1 r}}\right.\kern-0pt} \!\lower0.7ex\hbox{$r$}}}} ,$$

where $r$ is the ratio between the length of a specific linkage group and the length of the genome considered for QTL detection (1728 cM). The 95% confidence intervals of the QTL locations were estimated by LOD drop-off. In practice, the bounds of each interval were the two locations at which the likelihood was equal to the maximum likelihood minus 3.84 (=$\chi^{2} \left( {1, 0.05} \right)$) [24]. The QTL effect ($\alpha$) was expressed in phenotypic standard deviation units (${\text{SD}}$), and estimated as: $\alpha = \frac{1}{\text{SD}} \times \frac{1}{n}\mathop \sum \nolimits_{i = 1}^{n} \left| {\alpha_{i} } \right|$, where SD is the phenotypic standard deviation, $n$ the number of sires and $\alpha_{i}$ the effect of the within-sire $i$th QTL allele [25].

QTL detections were first carried out for phenotypic traits (QTL) and proteomic traits (pQTL) on a single-trait basis. For all confidence intervals of single phenotypic QTL and pQTL that overlapped, multi-trait QTL analyses, usually via a two-trait approach (2t-QTL), were performed [26] in order to identify possible pleiotropic effects between the phenotypes and liver protein variations. In addition, when a protein was identified for several spots each having a QTL on the same linkage group, the two-trait approach was also implemented to check whether the overall change in this protein improved the QTL.

To distinguish between pleiotropy and close linkage in 2t-QTL results, we performed the CLIP (Close LInkage versus Pleiotropy) test proposed by David et al. [27]. The CLIP test considers that under the assumption of pleiotropy (H0), the pattern of the SNP effects when moving along the tested genomic region should be similar for both traits, whereas under the close-linked QTL assumption (H1) it should be different.

### Graph inference

To aid interpretation, data from the CLIP test showing pleiotropy were transformed into graphs using the Gephi 0.9.1 software [28]. Gephi is an open-source and free visualization and exploration platform for all kinds of graphs. Weight was added to links for which pleiotropy was not rejected, and spatial statistics were used to identify nodes of importance in the graph. For example, nodes with a strong betweenness related to centrality were essential for the stability of the graph, and without such nodes the graph is disrupted [29].

### Functional annotation

To determine the biological relevance of the results, the Ingenuity Pathway Analysis (IPA, QIAGEN, Redwood City, www.qiagen.com/ingenuity) software was used to perform enrichment analysis (biological functions and canonical pathways), to construct bibliographic networks and regulation networks based on the identification of potential upstream regulators. Since IPA uses gene names, protein names were changed for gene names when necessary. Briefly, IPA constructed networks based on bibliographic data in which the edges were obtained from biological links such as receptor-ligand interactions, enzyme activity on another protein, or a transcriptional factor that activates the expression of targeted genes. IPA proposed the most probable network with an associated score. The final graph was reconstructed from the proposed IPA network with the best score using the PathDesigner function and also included information on some of the most significant canonical pathways and biological functions, as well as information from an interesting pleiotropic QTL.

## Results

### Single-trait QTL analysis

A total of 10,500 single-trait QTL analyses (28 chromosomes with 326 protein quantification traits and 49 phenotypic traits) were performed. We detected 287 significant pQTL at the 5% chromosome-wide threshold and in 176 cases, the protein was successfully identified (see Additional file 2: Table S2). We also detected 80 significant QTL at the 5% chromosome-wide threshold for the phenotypic traits (see Additional file 3: Table S3). Of these QTL and pQTL, 45 and 21 were significant at the 1% chromosome-wide threshold for the proteomic and phenotypic traits, respectively. At the genome-wide level, six phenotypic and five proteomic traits reached the 5% threshold and one phenotypic and two proteomic traits reached the 1% threshold.

**Fig. 4:**
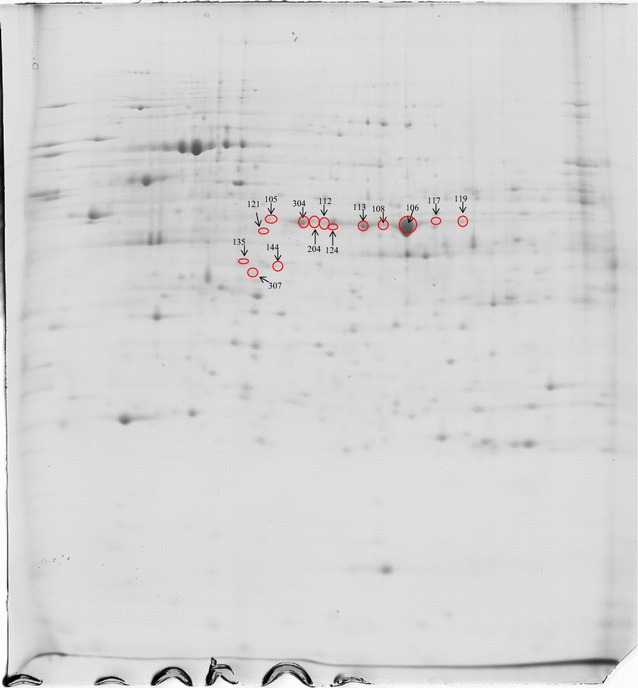
Alpha-enolase spot localization on gel electrophoresis

The 176 pQTL were located across the 28 linkage groups of the genetic map (see Additional file 2: Table S2). On average, we detected 6.3 (SD = 3.2) pQTL per chromosome. Surprisingly, the number of pQTL tended to be smaller on the macro-chromosomes (APL1 to APL10 and APLZ) than on the micro-chromosomes, with mean numbers of 5.7 ± 2.3 and 6.8 ± 3.7, respectively. In general, the allelic substitution effect for these 176 pQTL was low (on average 0.42 ± 0.17 of the standard deviation for the considered trait and ranged from 0.25 to 1.53) and the confidence intervals of the QTL were relatively large, with an average of 20 cM. Some chromosomes were considered particularly noteworthy because they harbored very significant pQTL. On APL18 and 24, we observed two pQTL significant at the 1% genome-wide significance level, respectively for alpha-enolase (ENO1) and fatty acid synthase (FASN) (see Additional file 2: Table S2). Moreover, the allele substitution effect at the QTL for ENO1 reached 1.5 standard deviations, which was one of the most important effects among all the pQTL detected. At the 5% genome-wide threshold, we observed two pQTL on APL10 for 3-hydroxyisobutyryl-CoA hydrolase (HIBCH) and persulfite dioxygenase ETHE1 (ETHE1), and a single pQTL on APL1 for peroxiredoxin 3 (PRDX3), on APL7 for apolipoprotein A1 (APOA1) and on APL21 for carbonic anhydrase 2 (CA2). Other chromosomes displayed a large number of pQTL at the 5% chromosome-wide threshold, i.e. APL24, 25 and 29 harboured more than 10 pQTL, and APL18 carried 15 pQTL. In a few cases, single protein spots mapped to several pQTL, i.e. spots 116 (proteasome 26S subunit ATPase 3—PSMC3), 124 (ENO1), 230 (phosphoglycerate mutase 1—PGAM1) and 301 (guanosine diphosphate dissociation inhibitor 2—GDI2) mapped to four pQTL each at the 5% chromosome-wide threshold. In addition, several spots can correspond to different co-existing forms of the same protein, due to variations of the electric charge and/or molecular weight resulting from post-translational modifications. Each of the spots for a given protein may map to one or several pQTL, which may or may not co-localize. For example, this was the case for ENO1, for which 14 protein spots were identified on the gels (Fig. 4), among which 12 mapped to up to 17 pQTL.

**Fig. 5:**
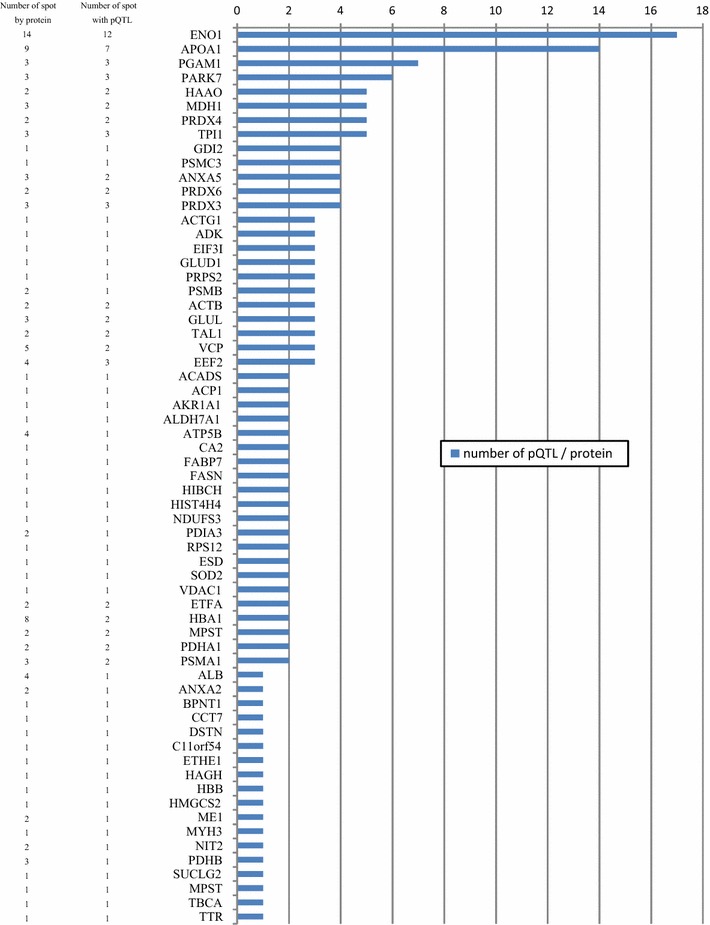
Proteins with pQTL detected in different spots. Protein descriptions are in Additional file 1: Table S1

Likewise, 14 pQTL were detected for APOA1, which displayed nine protein spots on the gel (Fig. 5). Five to seven pQTL were observed for phosphoglycerate mutase 1 (PGAM1), the putative Parkinson disease autosomal recessive early onset 7 variant 1 (PARK7), triose phosphate isomerase (TPI1), peroxiredoxin 4 (PRDX4), malate dehydrogenase 1 (MDH1) and 3-hydroxyanthranilate 3,4-dioxygenase (HAAO). For 86% of the 176 pQTL, the gene corresponding to the identified protein was mapped to the chicken genome, which allowed us to assign it to a duck chromosome. In 148 cases, the gene was localized on a chromosome other than that carrying the pQTL, which allowed us to unambiguously define it as a trans-pQTL [30]. In six cases, the gene was localized on the same chromosome than that carrying the pQTL, i.e. for S-formylglutathione hydrolase (ESD) on APL1, MDH1 on APL3, glutamate deshydrogenase (GLUD1) and PGAM1 on APL6, annexin A5 (ANXA5) on APL11 and alpha-aminoadipic semialdehyde dehydrogenase (ALDH7A1) on APLZ.

Regarding the 80 phenotypic QTL, the average allele substitution effect was equal to 0.42 ± 0.12 standard deviations (ranging from 0.26 to 1.04), with a confidence interval of about 17 cM (see Additional file 3: Table S3). These QTL are located on 24 of the 28 linkage groups of the genetic map. Some linkage groups seemed to be specific to a type of trait, with QTL for similar traits mapping very close to one another. Thus, five QTL related to growth mapped to APL3 at position 0.44 M, whereas the QTL detected on APL2 and 23 were more specific to liver composition and its melting rate, at 0.64 and 0 M on APL2 and APL23, respectively. Moreover, on APL2 we detected a QTL for melting rate that reached the 1% genome-wide threshold. At the 5% genome-wide threshold, six QTL were noteworthy: for liver protein content and liver weight on APL2, for bodyweight at ages 28 and 42 days on APL3, for weight gain during the overfeeding period on APL9 and for liver yellowness on APL18.

### Two-trait QTL analysis

Following single-trait QTL analyses, we conducted 290 protein-phenotype two-trait analyses (2t-QTL) when the confidence intervals for pQTL and phenotypic QTL overlapped. Among these, the *P* values for 66 2t-QTL were more significant than the *P* value of the strongest QTL using the single-trait approach (Table 2). The CLIP test was performed for all 66 2t-QTL and provided results for 37 of them, whereas for the 29 other 2t-QTL, the CLIP test did not converge mainly due to the lack of variability for traits such as plasma level of cholesterol, and to a lesser extent plasma level of triglycerides at the beginning of the overfeeding period, and corticosterone levels before stress.

**Table 2:** Sixty-six significant two-trait QTL for protein quantification and zootechnical traits

APL^a^	Traits					Threshold			CLIP test	
	Protein^b^	Phenotype	Location (cM)^c^	LRTx	Confidence interval^d^	biQTL *P* value (%)	UniQTL1 *P* value (%)	UniQTL2 *P* value (%)	Number of markers	Hyp^e^
3	MDH1 (179)	BW12	61	27.155	48–69	0.66	2.09	3.44	29	CL
5	ALDH7A1 (300)	BWG12-42	0	23.653	0–2	0.14	0.68	4.18	1	CL
5	ACTB (159)	BWG12-42	1	18.018	0–2	1.05	3.06	4.18	1	CL
5	VCP (283)	BWG12-42	1	20.996	0–2	0.38	3.30	4.18	1	CL
5	VCP (283)	AFW	1	20.125	0–2	0.61	3.30	4.84	1	CL
6	PRDX6 (231)	LColC	1	25.817	0–18	0.44	4.41	2.18	8	–
6	MDH1 (169)	LColC	14	27.870	0–22	0.15	0.33	2.18	8	–
6	GLUD1 (319)	LL*	36	20.153	10–48	2.02	3.80	2.54	8	PL
7	PGAM1 (230)	TSW	37	33.005	32–66	0.03	4.33	0.80	12	–
7	AKR1A1 (149)	TSW	42	38.135	33–45	0.03	0.98	0.80	12	CL
7	PGAM1 (230)	BWbeg	61	26.099	44–70	0.61	4.33	4.54	12	–
7	GLUD1 (319)	TSW	63	32.601	53–70	0.11	4.16	0.80	12	–
7	GLUD1 (319)	BWbeg	65	27.396	58–70	0.40	4.16	4.54	12	–
7	APOA1 (270)	TSW	69	38.558	61–70	0.01	0.06	0.80	12	PL
7	APOA1 (270)	BWbeg	69	38.103	62–70	0.04	0.06	4.54	12	CL
8	PRPS2 (192)	Menergy	0	23.879	0–9	1.20	1.25	2.72	10	CL
8	PRDX4 (237)	Menergy	5	22.179	0–13	1.84	4.65	2.72	10	CL
9	FABP7 (318)	BW12	21	22.066	11–35	1.66	3.97	2.45	11	PL
9	HISTH4 (315)	pmSFW	80	23.876	49–83	0.81	3.84	4.06	11	CL
10	HIBCH (151)	GLU 2nd M	12	31.044	4–21	0.03	0.03	0.81	9	PL
12	PRDX6 (226)	CHO 2nd M	61	23.217	37–66	1.00	3.04	3.33	26	CL
14	PDHA1 (133)	Mb*	0	19.704	0–9	2.33	3.75	3.67	14	–
14	ADK (136)	Mb*	0	19.395	0–8	2.71	3.15	3.67	14	–
14	HIBCH (151)	TG 2nd M	40	21.241	27–69	1.34	2.93	2.44	14	–
14	HIBCH (151)	CHO 20th M	69	27.010	45–69	0.17	2.93	4.90	14	–
15	PSMC3 (116)	CHO 2nd M	29	19.869	11–40	2.63	4.38	4.94	6	CL
15	MPST (188)	CHO 2nd M	33	19.938	22–48	2.58	4.16	4.94	6	PL
15	ENO1 (124)	CHO 2nd M	35	24.632	26–50	0.63	0.73	4.94	6	CL
15	ANXA5 (207)	CHO 2nd M	35	21.660	25–48	1.52	3.25	4.94	6	–
15	CCT7 (294)	MR	40	26.662	30–48	0.29	0.56	0.77	6	CL
15	ENO1 (124)	MR	43	28.102	30–52	0.19	0.73	0.77	6	PL
15	ENO1 (112)	MR	53	24.743	44–57	0.49	1.20	0.77	6	PL
18	PGAM1(325)	CortL	0	26.128	0–37	0.53	2.27	4.86	10	–
18	PGAM1 (232)	CortL	0	27.844	0–12	0.26	1.01	4.86	10	–
18	PRDX3 (257)	CortL	5	30.485	0–12	0.15	0.43	4.86	10	–
18	MDH1 (179)	CortL	11	28.325	0–15	0.16	1.40	4.86	10	–
18	GLUL (131)	CortL	13	28.171	8–16	0.32	1.07	4.86	10	–
18	VCP (285)	CortL	17	22.468	0–37	1.85	4.52	4.86	10	–
18	GDI2 (301)	CortL	18	27.892	15–27	0.35	1.34	4.86	10	–
19	PARK7 (263)	LProtC	0	19.718	0–21	1.95	2.45	4.37	6	CL
19	EIF3I (180)	LProtC	3	20.937	0–7	1.29	1.95	4.37	6	CL
19	EIF3I (180)	CHO 2nd M	4	26.900	0–7	0.19	1.95	3.31	6	–
19	PARK7 (263)	CHO 2nd M	4	24.653	0–8	0.37	2.45	3.31	6	–
19	ACP1 (274)	CHO 2nd M	4	25.729	0–6	0.29	2.00	3.31	6	–
19	EEF2 (306)	CHO 2nd M	4	20.905	0–20	1.12	4.69	3.31	6	–
19	EIF3I (180)	BW28	4	24.539	0–12	0.38	1.95	2.71	6	PL
19	ACP1 (274)	BW28	4	22.269	0–8	0.73	2.00	2.71	6	PL
19	EIF3I (180)	BWG12-28	4	22.948	0–16	0.62	1.95	3.39	6	PL
19	ACP1 (274)	BWG12-28	4	22.473	0–8	0.82	2.00	3.39	6	PL
19	PARK7 (263)	BWG12-28	6	23.640	0–20	0.46	2.45	3.39	6	–
19	PARK7 (263)	BW28	6	23.817	0–20	0.49	2.45	2.71	6	CL
19	EEF2 (306)	BWG12-28	16	22.160	0–20	0.83	4.69	3.39	6	–
19	EEF2 (306)	BW28	33	22.876	23–41	0.64	4.69	2.71	6	PL
21	ENO1 (112)	MFmax	46	18.664	41–57	3.72	5.00	4.84	12	CL
21	HAAO (196)	MFmax	46	20.570	41–55	2.04	2.12	4.84	12	CL
21	ALB (290)	MFmax	55	20.368	44–58	0.11	4.63	4.84	12	CL
22	BPNT1 (156)	BWG28-42	0	19.784	0–10	1.17	2.67	3.87	4	–
22	PRDX4 (309)	Lb*	0	23.820	0–4	0.28	0.83	0.98	4	PL
22	CA2 (227)	BWG28-42	9	19.204	0–10	1.63	3.26	3.87	4	–
22	CA2 (227)	Lb*	9	22.351	0–10	0.51	3.26	0.98	4	–
Z	GLUD1 (319)	GLU 2nd M	7	25.930	5–11	0.29	1.13	1.22	6	PL
Z	TPI1 (236)	MpHu	23	30.798	18–34	0.01	0.63	0.29	6	PL
Z	ENO1 (144)	MpHu	23	28.755	17–35	0.06	4.84	0.29	6	CL
Z	ALDH7A1 (300)	MpHu	30	28.204	22–35	0.11	4.99	0.29	6	–
Z	PSMA1 (212)	MpHu	32	30.809	31–35	0.05	1.14	0.29	6	–
Z	ATP5B (103)	MpHu	32	27.477	31–35	0.14	2.51	0.29	6	PL

Only 2t-QTL for which the *P* value is more significant than that of the stronger of the two QTL detected using the single-trait approach are reported in this table. These results were used to identify 16 assumed pleiotropic QTL

^a^Duck (*Anas platyrhynchos*) APL chromosome or linkage group

^b^Protein descriptions: see supplementary data

^c^Position on the genetic map in centiMorgans

^d^Confidence interval in centiMorgans

^e^CLIPtest: CL, close linkage; PL, pleiotropism; –, not tested

Among the 37 CLIP tests that provided results, the hypothesis of pleiotropy was not rejected for 16 2t-QTL. In particular, this was the case for: liver lightness and GLUD1 on APL6; thigh/shank weight and APOA1 on APL7; bodyweight at 12 days and fatty acid binding protein 7 (FABP7) on APL9; blood glucose at the beginning of the force-feeding period and HIBCH on APL10; blood cholesterol level at the beginning of the force-feeding period and 3-mercaptopyruvate sulfurtransferase (MPST) on APL15; melting rate and two spots for ENO1 on APL15; bodyweight at 28 days and eukaryotic translation initiation factor 3 subunit 1 (EIF3I), acid phosphatase 1 (ACP1), eukaryotic elongation factor 2 (EEF2) and bodyweight gain between 12 and 28 days of age and EIF3I and ACP1 on APL19; liver yellowness and PRDX4 on APL22; and ultimate muscle pH and ATP synthase subunit beta (ATP5B) and TPI1 and blood glucose at the beginning of the force-feeding period and GLUD1 on APLZ.

For 21 of the 37 two-trait analyses, we concluded that both QTL were in close linkage and rejected the hypothesis of pleiotropy. For example, although the *P* value of the 2t-QTL identified on APL21 for muscle maximal shear force and albumin protein (ALB) was clearly higher than those obtained in the single-trait QTL analysis, we concluded that these two QTL were linked but not pleiotropic. The same applied to thigh/shank weight and alcohol dehydrogenase (AKR1A1) on APL7, bodyweight gain between 12 and 42 days and valosin containing protein (VCP) on APL5, breast skin and subcutaneous fat weight and histone H4 (HIST4H4) on APL9, and bodyweight at 28 days and PARK7 on APL19.

**Fig. 6:**
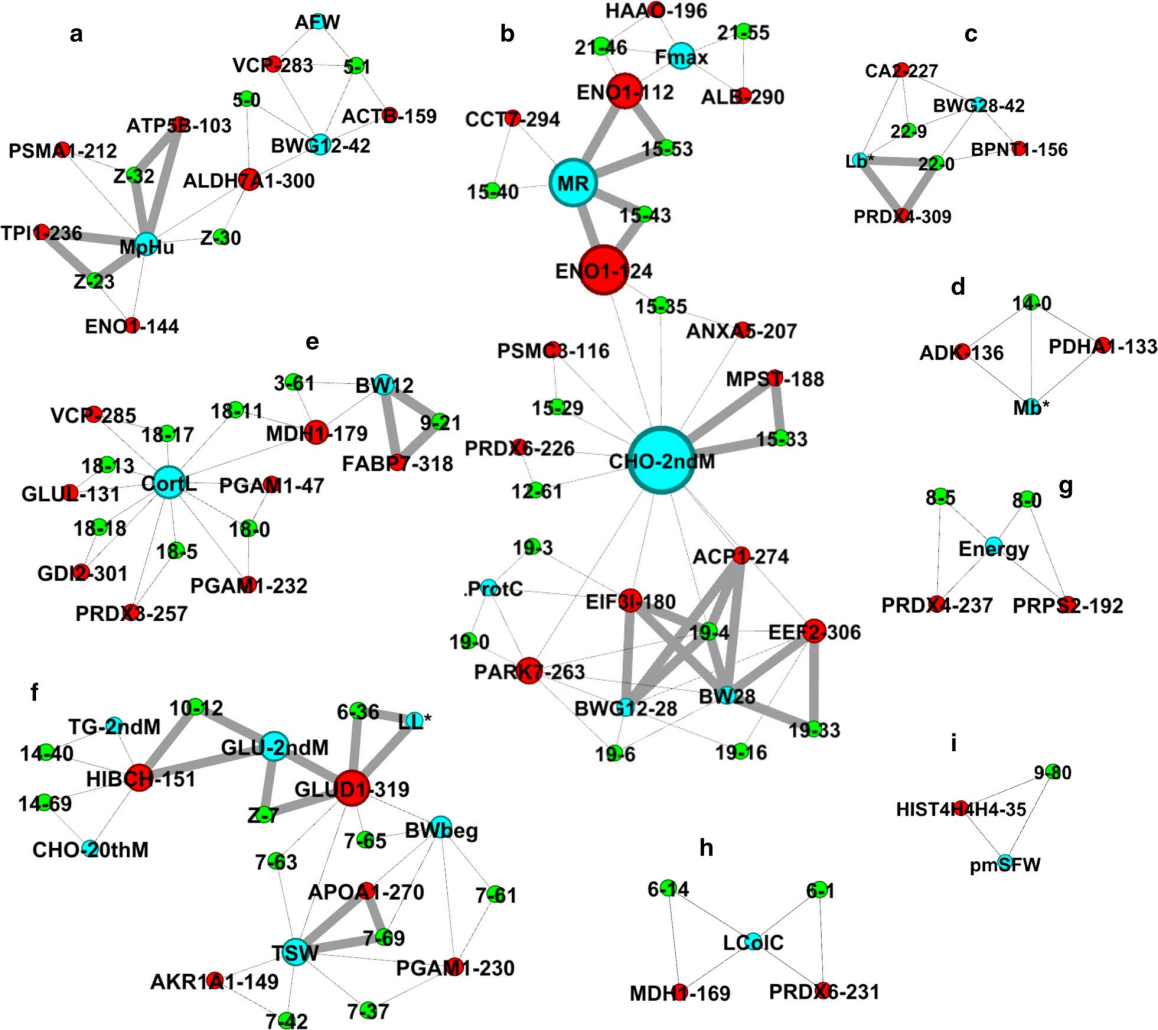
Graphs inferred from two-trait QTL detection with pleiotropy. These graphs are a representation of the data from Table 2. The chromosome locations are illustrated in green with the APL chromosome number and the location in cM. The phenotypic traits are in * blue* and the proteins are in * red* associated with the spot number. A weight is given for links (*in bold*) when a pleiotropic QTL was detected. The size of the nodes (proteins or phenotypes) is related to the betweenness (calculated by Gephi), i.e. an indicator of centrality that identifies the most important nodes within a graph

Graph inference using data from Table 2 resulted in nine graphs (Fig. 6) where pleiotropy is highlighted by weighted links (in bold on Fig. 6). This graphical representation of the data helps to detect pleiotropic traits and even possible epistatic events. It is interesting to observe how genomic regions that control many traits are represented as organized networks. For example, the QTL on APL10 (12 cM) and APLZ (7 cM) may both control plasma glucose levels (Fig. 6f). The ultimate pH of the *pectoralis major* muscle seems to be regulated by a QTL on APLZ (between 23 and 32 cM; Fig. 6a), which is associated with the abundance of ATP5B and TPI1, and to a lesser extent, with the abundance of ENO1, PSMA1 and ALDH7A1. Moreover, expression of ALDH7A1 is also controlled by another QTL on APL5 (0 cM). Likewise, even if no pleiotropy was detected, a region between 0 and 18 cM on APL18 seems to control the plasma levels of cortisol before stress, together with the abundance of PGAM1, PRDX3, MDH1, GLUD1, VCP and GDI2 (Fig. 6e).

Sixteen two-trait analyses were conducted for proteins that displayed several spots on 2D electrophoresis gels and for which QTL were detected on the same chromosome: eight for ENO1, four for APOA1, and one each for hemoglobin alpha (HBA1), PGAM1, glutamine synthetase (GLUL) and PRDX4. Among these analyses, the *P* values for six of the 2t-QTL were more significant than those for the strongest underlying single-trait QTL (Table 3), of which four involved ENO1. The CLIP test was performed for these six 2t-QTL and the pleiotropic hypothesis was not rejected in two cases: for ENO1, with spot numbers 124 and 307 on APL29 and spot numbers 112 and 124 on APL15. The phenotypic correlations between these ENO1 spots were quite low, about +0.24 for both spot pairs 112 and 124, and 124 and 307. Conversely, for APOA1 on APL24, we concluded that the QTL for spot numbers 174 and 262 were closely linked.

**Table 3:** Two-trait QTL detections of different spots of the same protein traits reveal two pleiotropic QTL

APL^a^	Protein^b^	Spot number^c^	Location (cM)^d^	LRTx	Confidence interval^e^	biQTL threshold (%)	UniQTL threshold (%)		CLIPTest^f^	
15	ENO1	112–124	51	26.220	41–57	0.41	0.73	1.20	6^g^	PL
18	PGAM1	232–325	0	31.645	0–11	0.12	1.01	2.27	10	–
24	APOA1	174–262	22	29.271	10–29	0.05	0.78	1.95	5	CL
25	ENO1	108–124	37	24.025	27–39	0.35	2.29	3.32	6	–
25	ENO1	108–304	38	28.653	34–39	0.14	2.29	0.40	6	–
29	ENO1	124–307	14	16.435	4–19	3.69	3.94	5.02	4	PL

^a^Duck (*Anas platyrhynchos*) APL chromosome or linkage group

^b^Protein descriptions: see supplementary data

^c^Spot number on the 2D gels

^d^Position on the genetic map in centiMorgans

^e^Confidence interval in centiMorgans

^f^CLIP test: CL, close linkage; PL, pleiotropy;–, nontested

^g^number of markers

### Biological analysis

**Fig. 7:**
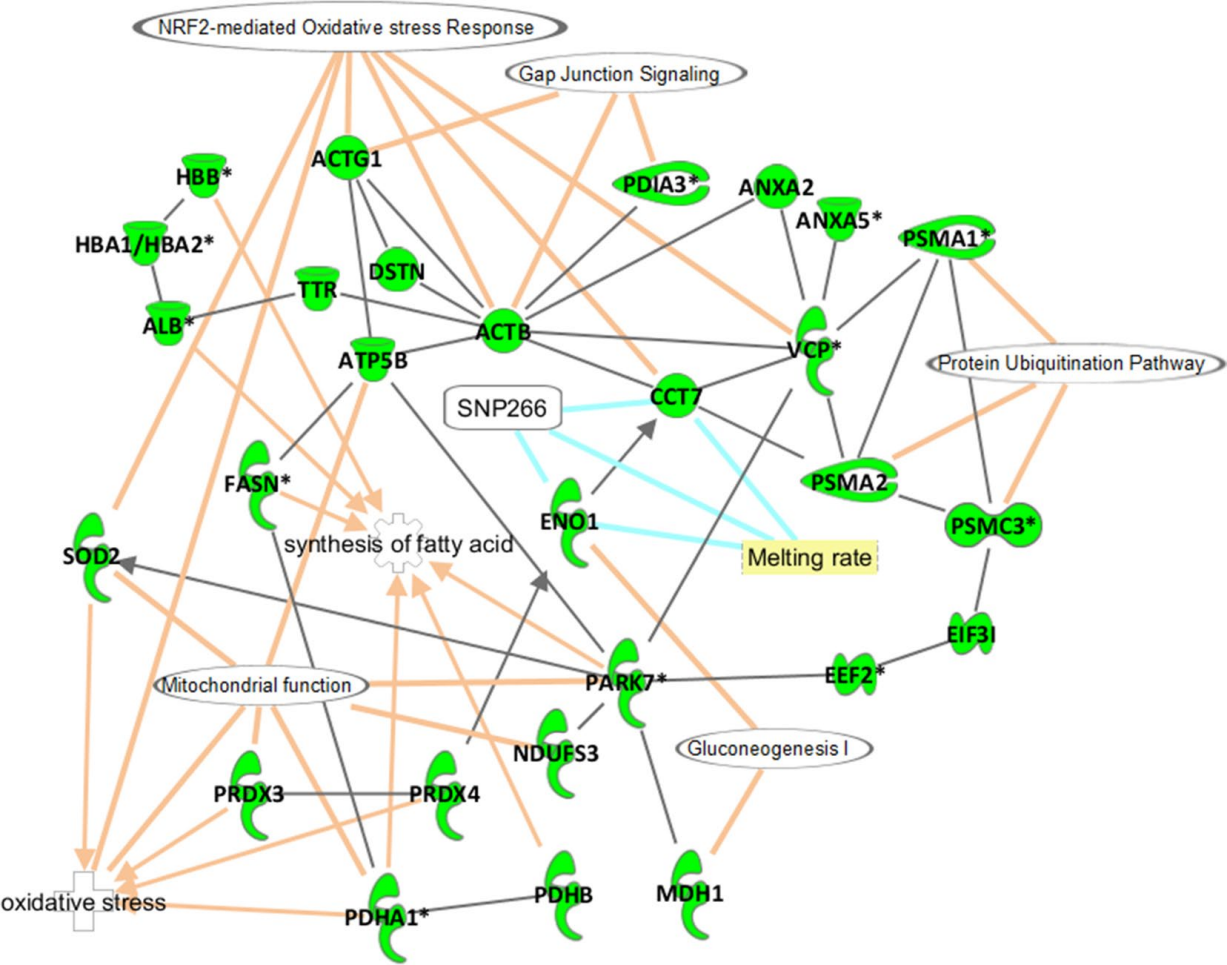
Biological network. This biological network was constructed with the proteins that are regulated by a QTL and associated with a significant enrichment score using Ingenuity Pathway Analysis software. Other information was added, such as some significant biological functions and canonical pathways (*links in orange*), proteins involved in cell viability are indicated with an *asterisk*. One trait related to liver function, i.e. melting rate, which is controlled by a pleiotropic QTL on APL15 (*links in blue*) was added

Among the 326 quantified spots, 190 were identified as corresponding to 97 unique proteins. Sixty-six proteins were regulated by at least one QTL (Fig. 5), i.e. two-thirds of the proteome were detected as being genetically regulated. We used the Ingenuity Pathway Analysis software to detect differences between the full proteome (all identified proteins) and the genetically-regulated proteome. Since not all proteins are recognized by IPA, 91 of the 97 unique proteins and 63 of the 66 regulated proteins were analysed. However, as expected, the processes were globally the same for the two proteome groups because proteins with pQTL form a subgroup of the complete proteome. For example, glycolysis and gluconeogenesis pathways were clearly enriched since all the proteins involved (ENO1, MDH1, ME1, PGAM1 and TPI1) were genetically regulated (Table 4 and Fig. 7). Likewise, the most significant pathway enriched in the genetically-regulated proteome is mitochondrial dysfunction for which seven of the nine identified proteins (PDHA1, PRDX3, SOD2, ATP5B, PARK7, VDAC1 and NDUFS3) had pQTL (Table 4 and Fig. 7).

**Table 4:** Functional enrichment analysis of canonical pathways between proteins with pQTL and the complete list of proteins

Ingenuity canonical pathways	Complete		pQTL		Proteins with pQTL in italic
	Regulated	Score^a^	Regulated	Score^a^	
Mitochondrial dysfunction	9/171 (5%)	7.22	7/171 (4%)	6.18	*PDHA1*, *PRDX3*, NDUFS1, *SOD2*, *ATP5B*, *PARK7*, GPX4, *VDAC1*, *NDUFS3*
Gluconeogenesis I	4/25 (16%)	5.40	4/25 (16%)	6.10	*ENO1*, *PGAM1*, *ME1*, *MDH1*
NRF2-mediated oxidative stress response	7/180 (4%)	4.85	6/180 (3%)	4.84	*AKR1A1*, *SOD2*, PRDX1, *ACTB*, *VCP*, *CCT7*, *ACTG1*
Glycolysis I	3/25 (12%)	3.76	3/25 (12%)	4.28	*ENO1*, *TPI1*, *PGAM1*
Acetyl-CoA biosynthesis I	3/7 (43%)	5.56	2/7 (29%)	3.76	*PDHA1*, DLAT, *PDHB*
LXR/RXR activation	5/121 (4%)	3.72	4/121 (3%)	3.36	*TTR*, *ALB*, *APOA1*, TF, *FASN*
FXR/RXR activation	5/126 (4%)	3.64	4/126 (3%)	3.29	
Caveolar-mediated endocytosis signaling	3/71 (4%)	2.43	3/71 (4%)	2.93	*ALB*, *ACTB*, *ACTG1*
Acute phase response signaling	5/169 (3%)	3.06	4/169 (2%)	2.82	*TTR*, *ALB*, *SOD2*, *APOA1*, TF
TR/RXR activation	3/85 (4%)	2.21	3/85 (4%)	2.70	*ENO1*, *FASN*, *ME1*
Tryptophan degradation X	4/23 (17%)	5.55	2/23 (9%)	2.69	ALDH2, *AKR1A1*, ALDH9A1, *ALDH7A1*
Ethanol degradation II	4/35 (11%)	4.79	2/35 (6%)	2.33	
Noradrenaline and adrenaline degradation	4/38 (11%)	4.65	2/38 (5%)	2.26	
Clathrin-mediated Endocytosis signaling	5/185 (3%)	2.88	4/185 (2%)	2.67	*ALB*, *APOA1*, TF, *ACTB*, *ACTG1*

Only the top 14 canonical pathways are in this table

Proteins regulated by a pQTL are in italics; pQTL may concern only a sub-list of the complete list of proteins identified by proteomic analysis

^a^Score corresponds to −log(*P* value)

The most significant biological function is the synthesis of purine nucleotides with nine of the 11 identified proteins regulated by a pQTL (Table 5). An interesting change between the full proteome versus the pQTL regulated proteome was observed for the function related to cell viability, which was found at the 26th and 41th positions for the complete proteome and at the 6th and 8th positions for the pQTL-regulated proteome. Finally, we implemented an integrative approach to reconstruct a biological network (Fig. 7) with PathDesigner (from Ingenuity) to highlight key results for the proteins with pQTL, for relevant pathways that are a priori regulated in our study, and for the liver melting rate trait, which is most important for producers since it is related to *foie gras* production.

**Table 5:** Functional enrichment analysis of biological functions between proteins with pQTL and the complete list of proteins

Diseases or function annotation (Ingenuity)	Complete			pQTL		
	*P* value	Number of molecules	Rank	*P* value	Number of molecules	Rank
Synthesis of purine nucleotide	9.97E−10	11	6	5.69E−09	9	1
Metabolism of nucleic acid component or derivative	7.89E−12	20	1	6.02E−09	14	2
Metabolism of dicarboxylic acid	2.21E−07	5	16	2.91E−08	5	3
Metabolism of nucleotide	2.07E−09	16	7	5.88E−08	12	4
Metabolism of hydrogen peroxide	2.63E−10	10	2	1.03E−07	7	5
Cell viability	6.29E−07	24	26	1.13E−07	20	6
Metabolism of nucleoside triphosphate	2.3E−08	9	11	3.14E−07	7	7
Cell viability of tumor cell lines	1.57E−05	16	41	3.45E−07	15	8
Catabolism of hydrogen peroxide	3.02E−10	6	4	3.83E−07	4	9
Biosynthesis of purine ribonucleotide	3.85E−07	7	21	6.28E−07	6	10
Synthesis of nucleotide	6.13E−07	12	25	6.86E−07	10	11
Biosynthesis of nucleoside triphosphate	5.98E−07	7	24	9.19E−07	6	12
Polymerization of protein	6.77E−06	11	35	1.06E−06	10	13
Fatty acid metabolism	1.46E−06	15	28	2.39E−06	12	14
Synthesis of acetyl-coenzyme A	1.64E−07	4	13	5.15E−06	3	15

Only the top 15 biological functions are shown according to Ingenuity analysis

## Discussion

### Sixty-eight percent of the proteins analyzed are partially controlled by QTL

Our group recently published the complete results of proteomic analyses carried out on 294 mule ducks [6] and showed that the abundance of 23 proteins was associated to three quality traits: liver weight, melting rate, and dry protein content. In the current study, our objective was to highlight the proteins for which abundance is partly genetically regulated. QTL detection was performed for all identified proteins and the issue of false positive results was raised. After Benjamini–Hochberg correction, even the most significant pQTL (for FASN on APL24) did not reach the 5% significance level since the adjusted *P* value was approximately 18%. However, regarding the power of our design and given the large number of QTL detections performed, we considered that the Benjamini–Hochberg correction was too drastic and decided to focus only on the more significant pQTL reaching the genome-wide significance threshold before correction. In this context, 66 out of the 97 unique proteins identified (68%) were regulated by at least one QTL.

### Most of the detected pQTL are trans-QTL

Amongst the 176 pQTL identified, the most significant were for APOA1 on APL7, ENO1 on APL18 and FASN on APL24. These three pQTL, together with more than 96% of the pQTL identified in this study, are located on a chromosome other than that carrying the gene coding for the protein analysed. This very high proportion of trans-acting pQTL suggests that the detected variations in protein quantity are generally not due to variations within their coding genes and the associated regulatory regions, such as promoters or enhancers. Such a high proportion of trans-acting eQTL was previously reported in humans, pigs [31] and rats, but cis-QTL usually have stronger effects [32]. Only six of the detected pQTL were putatively found on the same chromosome as that carrying the gene encoding the protein. However, at this point given the wide confidence intervals of these six pQTL, it is difficult to determine whether they are actually true cis-acting QTL. Until now, only three of the six genes coding for the six putative cis-pQTL are located on the duck genome assembly (*MDH1* on APL3, *ANXA5* on APL4 and *ALDH7A1* on APLZ). Only the pQTL for ALDH7A1 on APLZ is close to the gene coding for this protein, near the microsatellite marker CAM113 (Faraut T, personal communication). To explore this question further, a higher density genetic map and probably also addition of more families in the proteomics study are required to be able to observe a larger number of meiotic recombination events. The very high proportion of trans-acting QTL found in the current study could be due to the fact that protein levels are regulated by many more factors in addition to gene transcription. This is supported by the results of analysing separately spots corresponding to different forms of the same protein, which are very likely due to post-translational modifications.

### The most significant pQTL are related to fatty acid and amino acid metabolism, and glycolysis

Chromosome APL7, which harbours a pQTL for APOA1 that is significant at the 5% genome-wide level, seems to play an important role in regulating liver metabolism. APL7 also harbours phenotypic QTL for plasma glucose and cholesterol levels (Table 3) and for some weight traits (bodyweight before the overfeeding period, thigh and shank weight at slaughter). Indeed, APOA1 is involved in the transport of triglycerides from liver cells to adipose tissues by taking part in the formation of HDL (high density lipoprotein). Lagarrigue et al. [33] reported that the amounts of APOA1 mRNA were significantly larger in chickens from fat lines than from lean lines, which supported the hypothesis that it has a role in lipid transport and storage in birds. Szapacs et al. [34] showed that when APOA1 was exposed to oxidative changes, the formation of HDL and its exportation to the liver were altered. In chicken, GGA7, which is homoeologous to APL7, carries numerous QTL related to abdominal fat [35, 36]. Taken together, these results strengthen the hypothesis that chromosome APL7 is important in “fat” metabolism and further studies will be required to identify the genes that underlie the QTL and pQTL mapped to this chromosome. However, the only protein that we detected by 2D gel electrophoresis with a gene located on APL7 is HIBCH, which is involved in amino acid metabolism, but for which no QTL were mapped to APL7. The pleiotropic analysis with graphs (Fig. 6f) proposed interesting possible interactions between HIBCH, which is controlled by QTL on APL10 and APL14, and other QTL controlled by APL7.

Another strong pQTL was detected for ENO1 and mapped to APL18. Chromosome GGA17 is homoeologous to APL18 and harbours a QTL related to insulin levels in chickens [37]. This is interesting because both ENO1 levels and insulin levels are linked. Indeed, ENO1 is an enzyme of the glycolysis pathway where an increase in blood glucose level results in increased insulin synthesis and secretion by the pancreas leading to absorption of the glucose by the liver. Thus, glucose enters the glycolysis pathway to be transformed into pyruvate prior to fatty acid synthesis [38].

The strongest pQTL identified in this study was for FASN. In the liver, this enzyme plays a major role in lipid metabolism and lipid synthesis. Functional enrichment analysis identified FASN as playing a significant role in a pathway related to RXR activation (Table 4). RXR is a member of the nuclear receptor family of transcription factors and is closely related to nuclear receptors such as PPAR and FXR. The liver X receptors (LXR) are known to be important regulators of cholesterol, fatty acid, and glucose homeostasis. The pQTL related to FASN on APL24 co-localized with a QTL that affects plasma cholesterol levels but the significance of the 2t-QTL was lower, even if it is difficult to exceed the 1% genome-wide threshold in our design. It is interesting to note that other proteins for which the *P* value of the related QTL is between 1 and 5% on APL24, such as APOA1, PGAM1 or pyruvate dehydrogenase, are involved in lipid metabolism, which suggests that the locus on APL24 plays an important role in this metabolic pathway.

### Exploring the pleiotropic QTL

Previously, Gilbert and Leroy [26] demonstrated that, in the case of linked or pleiotropic QTL, combining phenotypic information from different traits could increase the precision of QTL mapping and possibly the power of single-trait analysis to detect QTL. Among the 66 CLIP tests that we performed (22% of the 2t-QTL), the pleiotropy hypothesis was not rejected for 16 of them, i.e. 5% of the initially performed two-trait analyses. This approach proved very effective for identifying likely pleiotropic QTL, and some of the 2t-QTL identified in this study are particularly interesting. Owing to the complexity of the data output, graphical representations were constructed (Fig. 6) to better illustrate the results and aid interpretation.

On chromosome APL15 (Fig. 6b), we were able to map several QTL and pQTL that were significant at the 1% chromosome-wide threshold around SNP266 among which one QTL was related to melting rate and two pQTL were related to CCT7 and ENO1 (spot 124), respectively. Two-trait analysis of CCT7 and liver melting rate revealed the presence of two closely-linked QTL, whereas two-trait analysis of ENO1 and melting rate revealed the presence of a QTL with a pleiotropic effect. Moreover, a second spot for ENO1 (spot 112) also appeared to act pleiotropically with melting rate, which means that a single locus affects both liver melting quality and ENO1 levels in spots 112 and 124, suggesting that the locus is involved in regulating glycolytic processes. Mapping duck SNP266 on the chicken genome showed that it is located in an intron of the *LMF1* (*lipoprotein maturation factor 1*) gene, which codes for a protein that is involved in the maturation of the lipoproteins before they leave liver cells [39]. These findings argue in favour of future studies on this region of APL15, to test this candidate gene and other nearby genes and identify the polymorphism that underlies the pleiotropic QTL.

On APLZ, a 2t-QTL was detected for GLUD1 and plasma glucose levels at the beginning of the force-feeding period, for which the pleiotropy hypothesis was not rejected. GLUD1 is a mitochondrial glutamate dehydrogenase 1 which plays a role in glutamine metabolism by converting l-glutamate into α-ketoglutarate. Although this enzyme is not directly involved in the lipid metabolism pathway, α-ketoglutarate is involved in the mitochondrial Krebs cycle by taking part in citrate synthesis, which is necessary for lipid synthesis in the liver. In humans, a syndrome called hyperinsulinism/hyperammonemia (HI/HA) could be due in part to mutations in the *GLUD1* gene [40] that increase the synthesis of α-ketoglutarate leading to an increase of insulin exocytosis in the pancreatic β-cells and consequently to an increase of glucose absorption by the liver. Since this mechanism occurs naturally after each meal, a pleiotropic QTL that, in ducks, affects both the abundance of GLUD1 and plasma glucose levels after feeding appears quite plausible. Since *GLUD1* is located on APL6, we can only speculate on the gene that is involved in this trans-acting two-trait QTL on APLZ.

A pleiotropic 2t-QTL for APOA1 and thigh weight mapped to APL7. Such an association of traits is unusual since the main peripheral tissue studied and linked with APOA1 is abdominal fat as explained previously. Although no association between abdominal fat and APOA1 could be tested since there is no known QTL for abdominal fat on APL7, our results are consistent with the fact that APOA1 has an important role in lipid exportation.

On APL19, the pleiotropic hypothesis was not rejected for the strong 2t-QTL between BW28 and EIF3I, ACP1 and EEF2, and between BWG12-28 and EIF3I and ACP1. EIF3I and EEF2 are proteins that act simultaneously on the ribosome to translate mRNA into protein. ACP1 is an enzyme that hydrolyses protein tyrosine phosphate. The association of such proteins with growth traits is interesting because growing cells and tissues require increased protein synthesis and it can be assumed that the genes underlying these QTL are involved in protein synthesis.

Of the 16 QTL for which a pleiotropic effect was detected, none mapped to the chromosomal position of the gene that encoded the protein tested. Therefore, we cannot directly identify the candidate protein or gene as suggested by Consoli et al. [41]. Interpretation of the 16 pleiotropic QTL detected in the current study is clearly more complex, since a variation in genotype probably affects a gene that modifies the metabolic pathway of the protein identified in the pQTL. Moreover, because of the low density of the duck genetic map and its incompletely annotated genome, it is impossible to formulate strong hypotheses on the genes or the gene functions that are highlighted by the different QTL. Nevertheless, the trait-protein associations that result in strong pleiotropic QTL are consistent and are the basis for preliminary explanations regarding the involved metabolic pathways.

## Conclusions

Through the identification of polymorphic genomic regions that are related to product quality and liver protein levels in overfed ducks, our aim was to better understand the metabolic mechanisms that are involved in the genetic variation of duck traits of economic value, such as fatty liver and breast (*magret*) quality. By analyzing co-localized phenotypic and proteomic QTL, we identified pleiotropic loci that affect metabolic pathways linked to glycolysis or lipogenesis. However, further investigation is required to confirm the protein biomarkers that were found to impact the genetic variability of phenotypic traits. Thus, the livers of these mule ducks are currently being phenotyped, both via transcriptomics and metabolomics approaches, in order to perform new QTL analyses based on “-omics” data and confirm or invalidate the metabolic pathways described in this paper. To further our understanding of the proteome, the proteomics approach presented here could be combined with mass spectrometry. Finally, once all “omics” QTL analyses are completed, it will be interesting to analyze QTL “hot spot” regions, which potentially harbor strong candidate genes with important regulatory functions in the liver.

## Additional files

**Additional file 1: Table S1.** List of identified protein spots for which QTL were detected. List of 104 protein spots with gene/protein symbol, protein name, spot number, accession number, Mascot score, number of amino acids, molecular weight and calculated isolectric point.

**Additional file 2: Table S2.** Single-trait QTL detection based on protein quantification. List of 176 pQTL (with successfully identified proteins) with APL chromosome number, protein name, spot number, QTL location (cM), maximum likelihood ratio, P-value, threshold reached, confidence interval, substitution effect and gene position on APL chromosome.

**Additional file 3: Table S3.** Single-trait QTL detection of zootechnical traits. List of 80 QTL with APL chromosome number, protein name, spot number, QTL location (cM), maximum likelihood ratio, P-value, threshold reached, confidence interval, substitution effect and gene position on APL chromosome.
