# Supplementary material for: Deciphering mechanisms underlying the genetic variation of general production and liver quality traits in the overfed mule duck by pQTL analyses
Source: Genet Sel Evol. 2017 Apr 19;49:38. doi: 10.1186/s12711-017-0313-6 (PMC5396126; doi:10.1186/s12711-017-0313-6)
Supplement: Supplementary file 1 — Additional file 1: Table S1. List of identified protein spots for which QTL were detected. List of 104 protein spots with gene/protein symbol, protein name, spot number, accession number, Mascot score, number of amino acids, molecular weight and calculated isolectric point. [file 12711_2017_313_MOESM1_ESM.docx]

**Table S1: List of identified protein spots for which QTLs were detected.**

#AA: number of amino acids; MW: molecular weight; calc. pI: calculated isolectric point

| Gene/Protein symbol | Protein name | N° | N° accession | Score Mascot | ΣCoverage | Σ# Peptides | # AAs | MW [kDa] | calc. pI |
| --- | --- | --- | --- | --- | --- | --- | --- | --- | --- |
| ACADS | Short-chain specific acyl-CoA dehydrogenase, mitochondrial | 147 | H0ZH79 | 763 | 24.58 | 8 | 415 | 44.3 | 8.66 |
| ACP1 | Low molecular weight phosphotyrosine protein phosphatase | 274 | Q5ZKG5 | 255.24 | 20.25 | 3 | 158 | 18.2 | 7.20 |
| ACTB | Beta-actin | 152 | F8UV49 | 669 | 53.56 | 9 | 295 | 33.2 | 5.52 |
| ACTB | Beta-actin | 159 | A6ZIB9 | 132 | 59.2 | 16 | 375 | 41.71 | 5.39 |
| ACTG1/ACTG | Gamma-actin | 211 | H9KYU9 | 171.8 | 11.39 | 3 | 316 | 35.2 | 5.25 |
| ADK | Adenosine Kinase | 136 | H0Z8F3 | 612 | 30.5 | 9 | 341 | 38 | 6.37 |
| AKR1A1 | Alcohol dehydrogenase | 149 | F1N9F8 | 129 | 12.54 | 5 | 327 | 37.1 | 7.8 |
| ALB | Albumine | 290 | I6U3L8 | 950.53 | 36.75 | 16 | 615 | 69.6 | 5.86 |
| ALDH7A1 | Alpha-aminoadipic semialdehyde dehydrogenase | 300 | H0YSW1 | 340 | 8.02 | 4 | 536 | 58.02 | 7.37 |
| ANXA2 | Annexin A2 | 171 | H0Z4R0 | 633.58 | 25.07 | 7 | 339 | 38.6 | 7.33 |
| ANXA5 | Annexin A5 | 201 | H0YUM4 | 764 | 27.53 | 8 | 316 | 35.4 | 5.20 |
| ANXA5 | Annexin A5 | 207 | H0YUM4 | 72 | 37.97 | 11 | 316 | 35.4 | 5.20 |
| APOA1 | Apolipoprotein A1 | 160 | O42296 | 697.34 | 52.65 | 15 | 264 | 30.6 | 5.54 |
| APOA1 | Apolipoprotein A1 | 174 | O42296 | 251 | 20.08 | 5 | 264 | 30.6 | 5.54 |
| APOA1 | Apolipoprotein A1 | 239 | O42296 | 306.3 | 17.8 | 4 | 264 | 30.6 | 5.54 |
| APOA1 | Apolipoprotein A1 | 240 | O42296 | 1398 | 63.64 | 19 | 264 | 30.6 | 5.54 |
| APOA1 | Apolipoprotein A1 | 245 | O42296 | 328 | 64.71 | 3 | 34 | 3.8 | 4.44 |
| APOA1 | Apolipoprotein A1 | 262 | O42296 | 294.99 | 25.38 | 6 | 264 | 30.6 | 5.54 |
| APOA1 | Apolipoprotein A1 | 270 | O42296 | 79 | 63.64 | 19 | 264 | 30.6 | 5.54 |
| APOA1 | Apolipoprotein A1 | 271 | O42296 | 372 | 63.64 | 19 | 264 | 30.6 | 5.54 |
| ATP5B | ATP synthase subunit beta, mitochondrial | 103 | Q5ZLC5 | 1759 | 35.27 | 15 | 533 | 56.59 | 5.87 |
| BPNT1 | 3'(2'),5'-bisphosphate nucleotidase 1 | 156 | F1P2D2 | 726.01 | 31.6 | 8 | 307 | 32.9 | 5.87 |
| C11orf54 | Ester hydrolase C11orf54 | 176 | H0ZRI8 | 171 | 22.15 | 4 | 316 | 34.8 | 6.24 |
| CA2 | Carbonic Anhydrase 2 | 227 | P07630 | 481 | 19.23 | 4 | 260 | 29 | 7.05 |
| CCT7 | T-complex protein 1 subunit eta | 294 | Q5ZJK8 | 1160.19 | 29.48 | 13 | 553 | 60.3 | 6.28 |
| DSTN | Destrin | 278 | H0Z6G7 | 254 | 27.44 | 4 | 164 | 18.4 | 7.91 |
| EEF2 | Elongation factor 2 | 166 | F1NFS0 | 264 | 5.13 | 4 | 858 | 95.3 | 6.74 |
| EEF2 | Eukaryotic Elongation Factor 2 | 198 | F1NFS0 | 515.73 | 9.32 | 6 | 858 | 95.3 | 6.74 |
| EEF2 | Eukaryotic Elongation Factor 2 | 306 | F1NFS0 | 352.69 | 8.28 | 5 | 858 | 95.3 | 6.74 |
| EIF3I | Eukaryotic translation initiation factor 3 subunit I | 180 | E1C6T8 | 435 | 28.62 | 8 | 325 | 36.47 | 5.64 |
| ENO1 | Alpha-enolase | 105 | P19140 | 283.16 | 17.28 | 5 | 434 | 47.2 | 6.80 |
| ENO1 | Alpha-enolase | 106 | P19140 |  | 65.21 | 28 | 434 | 47.2 | 6.80 |
| ENO1 | Alpha-enolase | 108 | P19140 | 2427 | 65.21 | 28 | 434 | 47.2 | 6.80 |
| ENO1 | Alpha-enolase | 112 | P19140 | 1358.48 | 39.63 | 12 | 434 | 47.2 | 6.80 |
| ENO1 | Alpha-enolase | 113 | P19140 | 5356 | 65.21 | 28 | 434 | 47.3 | 6.58 |
| ENO1 | Alpha-enolase | 117 | P19140 | 1399 | 29.72 | 11 | 434 | 47.2 | 6.80 |
| ENO1 | Alpha-enolase | 119 | P19140 | 1328.29 | 46.77 | 14 | 434 | 47.2 | 6.80 |
| ENO1 | Alpha-enolase | 124 | P19140 | 1483 | 39.17 | 11 | 434 | 47.2 | 6.80 |
| ENO1 | Alpha-enolase | 135 | P19140 | 827 | 65.21 | 28 | 434 | 47.2 | 6.80 |
| ENO1 | Alpha-enolase | 144 | P19140 | 416 | 15.44 | 4 | 434 | 47.2 | 6.80 |
| ENO1 | Alpha-enolase | 304 | P19140 | 4700 | 65.21 | 28 | 434 | 47.2 | 6.80 |
| ENO1 | Alpha-enolase | 307 | P19140 | 511.36 | 20.97 | 6 | 434 | 47.2 | 6.80 |
| ESD | S-formylglutathione hydrolase | 194 | G1NQ47 | 402 | 22.34 | 5 | 282 | 31.5 | 6.61 |
| ETFA | Electron transfer flavoprotein subunit alpha, mitochondrial | 202 | H0YZD0 | 320 | 22.67 | 5 | 322 | 34 | 7.77 |
| ETFA | Electron transfer flavoprotein subunit alpha, mitochondrial | 205 | G3UV65 | 266.39 | 15.12 | 3 | 291 | 30.6 | 7.33 |
| ETHE1 | Protein ETHE1, mitochondrial | 225 | H9L167 | 97 | 30.84 | 2 | 107 | 11.68 | 7.34 |
| FABP7 | Fatty Acid Binding Protein 7 | 318 | H0ZNK7 | 918.66 | 50 | 5 | 132 | 14.9 | 5.91 |
| FASN | Fatty acid synthase | 206 | B8XNP2 | 467 | 59.78 | 4 | 92 | 10.1 | 5.73 |
| GDI2 | Rab GDP dissociation inhibitor beta | 301 | F1NCZ2 | 712 | 42.26 | 12 | 433 | 49 | 5.73 |
| GLUD1/GDH1 | Glutamate Dehydrogenase 1, mitochondrial | 319 | P00368 | 122.86 | 6.56 | 3 | 503 | 55.7 | 8.28 |
| GLUL/GS | Glutamine Synthetase | 128 | G1MW53 | 460 | 22.79 | 7 | 373 | 42.2 | 6.98 |
| GLUL/GS | Glutamine Synthetase | 131 | G1MW53 | 466 | 24.93 | 8 | 373 | 42.2 | 6.98 |
| HIST4H4/H4-I | Histone H4 | 315 | G3UR23 | 161.88 | 44.78 | 3 | 67 | 7.6 | 10.48 |
| HAAO | 3-hydroxyanthranilate 3,4-dioxygenase | 193 | G1NCG5 | 124 | 12.8 | 3 | 289 | 33.6 | 6.32 |
| HAAO | 3-hydroxyanthranilate 3,4-dioxygenase | 196 | G1NCG5 | 108 | 9 | 3 | 289 | 33.6 | 6.32 |
| HAGH | Hydroxyacylglutathione hydrolase, mitochondrial | 229 | Q5ZI23-2 | 313.72 | 15 | 3 | 260 | 29 | 6.90 |
| HBA1/HBA | Hemoglobin Alpha | 139 | C7EM33 | 223.68 | 53.74 | 7 | 147 | 16.4 | 8.63 |
| HBA | Hemoglobin Alpha | 286 | C7EM33 | 446.61 | 48.3 | 6 | 147 | 16.4 | 8.63 |
| HBB | Hemoglobin Beta | 264 | P08261 | 137.68 | 30.14 | 3 | 146 | 16.2 | 8.84 |
| HIBCH | 3-hydroxyisobutyryl-CoA hydrolase, mitochondrial | 151 | G1N3F5 | 200 | 7.49 | 3 | 387 | 42.9 | 7.80 |
| HMGCS2 | Hydroxymethylglutaryl-CoA synthase | 302 | G1MZD6 | 571 | 13.07 | 6 | 505 | 55.1 | 7.36 |
| MDH1 | Malate dehydrogenase | 169 | G1N071 | 382 | 23.65 | 6 | 334 | 36.5 | 7.36 |
| MDH1 | Malate déhydrogénase 1 | 179 | G1N071 | 309.43 | 16.77 | 4 | 334 | 36.5 | 7.36 |
| ME1 | Malic enzyme | 293 | G1NLV4 | 162 | 10.05 | 4 | 557 | 62 | 7.06 |
| MPST | Sulfurtransferase | 188 | E1C8D8 | 486.62 | 24.91 | 5 | 293 | 32.8 | 6.01 |
| MPST | Sulfurtransferase | 189 | H9L016 | 32815 | 23.6 | 4 | 267 | 29.8 | 5.40 |
| MYH3/MYH1B | Myosin, Heavy Chain 3, Skeletal Muscle, Embryonic/Myosin-3 | 177 | F1ND26 | 1516 | 18.07 | 28 | 1942 | 223.11 | 5.90 |
| NDUFS3 | NADH dehydrogenase iron-sulfur protein 3 mitochondrial | 228 | F1ND23 |  | 30.38 | 6 | 237 | 27.3 | 5.91 |
| NIT2 | Nitrilase homolog 2 | 210 | F1NP29 | 164.38 | 9.19 | 3 | 283 | 31.3 | 7.05 |
| PARK7 | Protein deglycase DJ1 | 263 | B5G3Z6 | 220 | 21.69 | 5 | 189 | 19.95 | 6.79 |
| PARK7 | Protein deglycase DJ1 | 265 | B5G3Z6 | 323 | 21.69 | 5 | 189 | 19.95 | 6.79 |
| PARK7 | Protein deglycase DJ1 | 269 | B5G3Z6 | 323 | 21.69 | 5 | 189 | 19.95 | 6.79 |
| PDHA1 | Pyruvate Dehydrogenase E1 component subunit alpha | 133 | G1NP97 |  | 29.62 | 10 | 395 | 44.17 | 7.77 |
| PDHB | Pyruvate Dehydrogenase E1 component subunit beta, mitochondrial | 185 | G1N8K4 | 1743 | 41.92 | 11 | 334 | 36.1 | 5.33 |
| PDHB | Pyruvate Dehydrogenase E1 component subunit beta, mitochondrial | 190 | G1N8K4 | 708.61 | 31.74 | 8 | 334 | 36.1 | 5.33 |
| PDIA3 | Protein disulfide-isomerase A3 | 297 | H0ZIZ6 | 802.1 | 31.17 | 16 | 494 | 55.1 | 5.90 |
| PGAM1 | Phosphoglycerate mutase 1 | 230 | Q5ZLN1 | 551 | 31.89 | 6 | 254 | 28.9 | 7.49 |
| PGAM1 | Phosphoglycerate mutase 1 | 232 | Q5ZLN1 | 281 | 18.9 | 4 | 254 | 28.9 | 7.49 |
| PGAM1 | Phosphoglycerate mutase 1 | 325 | Q5ZLN1 | 953 | 36.61 | 10 | 254 | 28.88 | 7.49 |
| PRDX3 | Peroxiredoxin 3 | 256 | E1BR10 | 177 | 19.46 | 3 | 257 | 28 | 8.54 |
| PRDX3 | Peroxiredoxin 3 | 257 | H0ZLJ2 | 167.62 | 17.17 | 3 | 198 | 21.9 | 7.02 |
| PRDX3 | Peroxiredoxin 3 | 259 | H0ZLJ2 | 141 | 15.15 | 3 | 198 | 21.87 | 7.02 |
| PRDX4 | Peroxiredoxin 4 | 237 | H0ZAF6 | 205 | 25.52 | 4 | 192 | 21.8 | 6.20 |
| PRDX4 | Peroxiredoxin 4 | 309 | F1NNS8 | 502.53 | 35.07 | 8 | 288 | 32.1 | 8.40 |
| PRDX6 | Peroxiredoxin 6 | 226 | F1NBV0 | 645 | 43.75 | 8 | 224 | 25.1 | 6.38 |
| PRDX6 | Peroxiredoxin 6 | 231 | F1NBV0 | 1703 | 43.75 | 8 | 224 | 25.1 | 6.38 |
| PRPS2 | Ribose-phosphate pyrophosphokinase | 192 | E1BRQ0 | 483.71 | 40.31 | 9 | 325 | 35.6 | 6.68 |
| PSMA1 | Proteasome subunit Alpha 1 | 212 | H0ZDR3 | 238 | 18.08 | 3 | 260 | 29.2 | 6.54 |
| PSMA2 | Proteasome subunit Alpha 2 | 253 | B5G154 | 630 | 29.49 | 5 | 234 | 25.92 | 7.49 |
| PSMB | Proteasome subunit Beta | 215 | Q5ZJS9 | 122.35 | 19.9 | 3 | 201 | 21.3 | 8.94 |
| PSMC3 | 26S protease regulatory subunit 6A | 116 | F1NDQ1 | 146.81 | 18.70 | 5 | 385 | 43.0 | 5.69 |
| RPS12 | 40S ribosomal protein S12 | 321 | H0ZMV7 | 311.84 | 36.36 | 4 | 132 | 14.5 | 7.21 |
| SOD2/SODMn | Mn superoxide dismutase | 267 | B2M0J6 | 109.48 | 27.12 | 3 | 118 | 12.8 | 6.89 |
| SUCLG2 | Succinyl-coA ligase subunit beta | 127 | G3UR69 | 533.52 | 22.76 | 8 | 413 | 44.8 | 6.06 |
| TAL1/TAL | Transaldolase | 163 | G1MXK0 | 336 | 24.02 | 7 | 337 | 37.5 | 7.47 |
| TAL1/TAL | Transaldolase | 164 | G1MXK0 | 770 | 32.34 | 10 | 337 | 37.5 | 7.47 |
| TBCA | Tubulic-specific chaperone A | 312 | G1MZT0 | 302.77 | 48.35 | 6 | 91 | 10.8 | 4.88 |
| TPI1/TPI | Triose Phosphate Isomerase | 236 | Q70I43 | 915 | 67.3 | 11 | 211 | 22.6 | 6.64 |
| TPI1/TPI | Triose Phosphate Isomerase | 242 | Q70I43 | 936.8 | 65.88 | 10 | 211 | 22.6 | 6.64 |
| TPI1/TPI | Triose Phosphate Isomerase | 243 | Q70I43 | 1200 | 67.3 | 11 | 211 | 22.6 | 6.64 |
| TTR | Transthyretin | 324 | Q2LDE6 | 219.74 | 45.33 | 4 | 150 | 16.3 | 5.24 |
| VCP | Valosin Containing Protein | 283 | Q5ZMU9 | 6502 | 64.52 | 40 | 806 | 89.3 | 5.26 |
| VCP | Valosin Containing Protein | 285 | Q5ZMU9 | 486 | 64.52 | 40 | 806 | 89.3 | 5.26 |
| VDAC1 | Voltage-dependent anion-selective channel protein 1 | 216 | G1N8Q2 | 872 | 53.58 | 12 | 293 | 31.8 | 8.54 |
