# Supplementary material for: Deciphering mechanisms underlying the genetic variation of general production and liver quality traits in the overfed mule duck by pQTL analyses
Source: Genet Sel Evol. 2017 Apr 19;49:38. doi: 10.1186/s12711-017-0313-6 (PMC5396126; doi:10.1186/s12711-017-0313-6)
Supplement: Supplementary file 2 — Additional file 2: Table S2. Single-trait QTL detection based on protein quantification. List of 176 pQTL (with successfully identified proteins) with APL chromosome number, protein name, spot number, QTL location (cM), maximum likelihood ratio, P-value, threshold reached, confidence interval, substitution effect and gene position on APL chromosome. [file 12711_2017_313_MOESM2_ESM.docx]

**Table S2: single-trait QTL detection based on protein quantification.**

| **APL^1^** | **Identification^2^** | **N°^3^ spot** | **Location^4^**  **(cM)** | **LRTx** | ***P*-value** | **Threshold^5^** | | | **Confidence interval** | | **Subst. effect.** | | **Gene^6^** | |
| --- | --- | --- | --- | --- | --- | --- | --- | --- | --- | --- | --- | --- | --- | --- |
| 1 | ESD | 194 | 41 | 17.50 | 2.40% | - |  | 32-61 | | 0.38 | | APL1 | |  |
| **1** | **EEF2** | **166** | **94** | **20.03** | **0.89%** | *●* |  | **81-99** | | **0.43** | | APL29 | |  |
| 1 | MPST | 189 | 153 | 16.28 | 3.80% | - |  | 141-186 | | 0.40 | | - | |  |
| **1** | **PRDX3** | **259** | **200** | **22.88** | **0.27%** | *●●* | ‡ | **194-212** | | **0.45** | | APL6 | |  |
| 2 | DSTN | 278 | 74 | 16.20 | 2.64% | - |  | 71-75 | | 0.34 | | APL3 | |  |
| 2 | ETFA | 202 | 92 | 15.67 | 3.25% | - |  | 78-97 | | 0.37 | | APL10 | |  |
| 2 | MPST | 189 | 93 | 14.47 | 4.98% | - |  | 82-99 | | 0.40 | | - | |  |
| 3 | ACTB | 152 | 0 | 14.38 | 4.92% | - |  | 0-19 | | 0.45 | | APL10 | |  |
| 3 | HAGH | 229 | 0 | 14.73 | 4.32% | - |  | 0-13 | | 0.45 | | APL15 | |  |
| 3 | NDUFS3 | 228 | 4 | 15.58 | 3.16% | - |  | 0-16 | | 0.41 | | APL5 | |  |
| 3 | APOA1 | 271 | 39 | 16.38 | 2.33% | - |  | 22-62 | | 0.32 | | APL25 | |  |
| 3 | MDH1 | 179 | 55 | 16.66 | 2.09% | - |  | 44-70 | | 0.38 | | APL3 | |  |
| 3 | ENO1 | 124 | 64 | 15.10 | 3.76% | - |  | 33-71 | | 0.41 | | APL22 | |  |
| 3 | ADK | 136 | 72 | 15.08 | 3.79% | - |  | 59-85 | | 0.38 | | APL6 | |  |
| 3 | APOA1 | 245 | 72 | 14.89 | 4.07% | - |  | 68-91 | | 0.40 | | APL25 | |  |
| **3** | **TAL1** | **163** | **81** | **18.77** | **0.92%** | *●* |  | **73-100** | | **0.42** | | - | |  |
| **4** | **AKR1A1** | **149** | **0** | **19.04** | **0.33%** | *●●* |  | **0-17** | | **0.45** | | APL8 | |  |
| 4 | APOA1 | 240 | 23 | 13.33 | 3.27% | - |  | 0-39 | | 0.36 | | APL25 | |  |
| 4 | ACTG1 | 211 | 38 | 15.03 | 1.66% | - |  | 25-56 | | 0.33 | | APL10 | |  |
| 4 | SOD2 | 267 | 38 | 13.89 | 2.59% | - |  | 0-43 | | 0.38 | | - | |  |
| 4 | APOA1 | 174 | 59 | 15.08 | 1.62% | - |  | 50-73 | | 0.42 | | APL25 | |  |
| **4** | **EIF3I** | **180** | **93** | **16.74** | **0.83%** | *●* |  | **91-94** | | **0.50** | | APL24 | |  |
| **4** | **PRDX3** | **256** | **94** | **19.00** | **0.34%** | *●●* |  | **92-94** | | **0.44** | | APL6 | |  |
| **5** | **ALDH7A1** | **300** | **0** | **13.74** | **0.68%** | *●* |  | **0-2** | | **0.37** | | APL Z | |  |
| 5 | VCP | 283 | 1 | 9.92 | 3.30% | - |  | 0-2 | | 0.31 | | APL Z | |  |
| 5 | ACTB | 159 | 2 | 10.10 | 3.06% | - |  | 0-2 | | 0.34 | | APL10 | |  |
| 6 | PRDX6 | 231 | 6 | 10.29 | 4.41% | - |  | 0-25 | | 0.43 | | APL8 | |  |
| 6 | ETFA | 205 | 7 | 9.94 | 4.98% | - |  | 0-25 | | 0.46 | | APL10 | |  |
| **6** | **MDH1** | **169** | **16** | **16.73** | **0.33%** | *●●* |  | **0-27** | | **0.55** | | APL3 | |  |
| 6 | HAAO | 196 | 20 | 10.89 | 3.50% | - |  | 0-30 | | 0.40 | | APL3 | |  |
| 6 | HBB | 264 | 34 | 10.64 | 3.85% | - |  | 7-49 | | 0.45 | | - | |  |
| 6 | GLUD1 | 319 | 34 | 10.67 | 3.80% | - |  | 8-54 | | 0.37 | | APL6 | |  |
| **6** | **PGAM1** | **230** | **44** | **15.86** | **0.48%** | *●●* |  | **29-54** | | **0.50** | | APL6 | |  |
| 6 | PDHB | 190 | 54 | 13.15 | 1.43% | - |  | 37-54 | | 0.43 | | APL13 | |  |
| **7** | **AKR1A1** | **149** | **42** | **17.93** | **0.98%** | *●* |  | **40-45** | | **0.48** | | APL8 | |  |
| 7 | PGAM1 | 230 | 57 | 13.84 | 4.33% | - |  | 43-70 | | 0.35 | | APL6 | |  |
| 7 | GLUD1 | 319 | 64 | 13.95 | 4.16% | - |  | 43-70 | | 0.37 | | APL6 | |  |
| **7** | **APOA1** | **270** | **69** | **25.10** | **0.06%** | *●●●* | ‡ | **62-70** | | **0.52** | | APL25 | |  |
| 8 | PRPS2 | 192 | 0 | 16.27 | 1.25% | - |  | 0-9 | | 0.33 | | APL1 | |  |
| 8 | PRDX4 | 237 | 5 | 12.80 | 4.65% | - |  | 0-17 | | 0.35 | | APL1 | |  |
| 8 | PDIA3 | 297 | 19 | 14.17 | 2.80% | - |  | 0-25 | | 0.34 | | APL10 | |  |
| 8 | HBA1 | 286 | 30 | 13.74 | 3.31% | - |  | 16-41 | | 0.38 | | - | |  |
| 8 | ACTB | 152 | 40 | 15.01 | 2.03% | - |  | 2-64 | | 0.35 | | APL10 | |  |
| 8 | APOA1 | 160 | 45 | 12.78 | 4.69% | - |  | 21-64 | | 0.32 | | APL25 | |  |
| 8 | HBA1 | 139 | 53 | 14.39 | 2.56% | - |  | 38-64 | | 0.38 | | - | |  |
| 9 | HAAO | 193 | 8 | 12.47 | 3.87% | - |  | 2-39 | | 0.36 | | APL3 | |  |
| 9 | FABP7 | 318 | 17 | 12.40 | 3.97% | - |  | 0-31 | | 0.39 | | APL3 | |  |
| 9 | HIST4H4 | 315 | 24 | 12.49 | 3.84% | - |  | 0-47 | | 0.42 | | APL1 | |  |
| 10 | PSMC3 | 116 | 0 | 14.11 | 1.76% | - |  | 0-16 | | 0.41 | | APL5 | |  |
| 10 | APOA1 | 239 | 1 | 12.22 | 3.72% | - |  | 0-15 | | 0.30 | | APL25 | |  |
| **10** | **HIBCH** | **151** | **11** | **24.11** | **0.03%** | *●●●* | ‡ | **2-20** | | **0.52** | | APL7 | |  |
| 10 | PRDX4 | 237 | 28 | 12.17 | 3.79% | - |  | 17-39 | | 0.29 | | APL1 | |  |
| 10 | ACP1 | 274 | 36 | 13.33 | 2.40% | - |  | 33-40 | | 0.35 | | APL3 | |  |
| 10 | HIST4H4 | 315 | 36 | 12.48 | 3.37% | - |  | 19-40 | | 0.37 | | APL1 | |  |
| **10** | **ETHE1** | **225** | **39** | **21.08** | **0.11%** | *●●* | ‡ | **34-40** | | **0.45** | | - | |  |
| **11** | **ANXA5** | **201** | **9** | **16.67** | **0.75%** | *●* |  | **3-13** | | **0.81** | | APL11 | |  |
| **11** | TPI1 | **243** | **22** | **16.84** | **0.70%** | *●* |  | **0-25** | | **0.44** | | APL1 | |  |
| 12 | PRDX6 | 226 | 69 | 13.45 | 3.04% | - |  | 67-71 | | 0.31 | | APL8 | |  |
| 12 | APOA1 | 240 | 69 | 14.66 | 3.63% | - |  | 65-71 | | 0.38 | | APL25 | |  |
| **12** | **APOA1** | **245** | **114** | **16.44** | **0.99%** | *●* |  | **93-129** | | **0.55** | | APL25 | |  |
| 12 | HAAO | 193 | 129 | 15.46 | 1.41% | - |  | 112-155 | | 0.52 | | APL3 | |  |
| 12 | ESD | 194 | 148 | 14.29 | 2.21% | - |  | 122-155 | | 0.37 | | APL1 | |  |
| 12 | NIT2 | 210 | 151 | 13.88 | 2.57% | - |  | 118-155 | | 0.34 | | APL1 | |  |
| 12 | ENO1 | 117 | 155 | 13.26 | 3.26% | - |  | 133-155 | | 0.36 | | APL22 | |  |
| 12 | TAL1 | 164 | 155 | 13.17 | 3.37% | - |  | 133-155 | | 0.38 | | - | |  |
| 13 | ENO1 | 135 | 9 | 12.39 | 1.90% | - |  | 0-25 | | 0.39 | | APL22 | |  |
| 13 | TAL1 | 164 | 13 | 10.27 | 4.50% | - |  | 7-31 | | 0.34 | | - | |  |
| **13** | **VDAC1** | **216** | **47** | **15.92** | **0.43%** | *●●* |  | **28-53** | | **0.51** | | - | |  |
| 13 | PGAM1 | 230 | 60 | 10.41 | 4.25% | - |  | 26-74 | | 0.32 | | APL6 | |  |
| 14 | ADK | 136 | 4 | 12.78 | 3.15% | - |  | 0-16 | | 0.37 | | APL6 | |  |
| 14 | PDHA1 | 133 | 5 | 12.35 | 3.75% | - |  | 0-51 | | 0.32 | | - | |  |
| 14 | HIBCH | 151 | 52 | 12.97 | 2.93% | - |  | 26-69 | | 0.29 | | APL7 | |  |
| 15 | PSMC3 | 116 | 22 | 12.23 | 4.38% | - |  | 0-38 | | 0.34 | | APL5 | |  |
| **15** | **FASN** | **206** | **25** | **16.63** | **0.80%** | *●* |  | **0-37** | | **0.49** | | APL19 | |  |
| 15 | MPST | 188 | 33 | 12.37 | 4.16% | - |  | 6-55 | | 0.37 | | APL | |  |
| 15 | ANXA5 | 207 | 35 | 13.02 | 3.25% | - |  | 21-53 | | 0.40 | | APL4 | |  |
| **15** | **CCT7** | **294** | **38** | **17.52** | **0.56%** | *●* |  | **24-48** | | **0.46** | | APL4 | |  |
| **15** | **ENO1** | **124** | **43** | **16.85** | **0.73%** | *●* |  | **24-54** | | **0.46** | | APL22 | |  |
| 15 | ENO1 | 112 | 55 | 15.59 | 1.20% | - |  | 46-57 | | 0.36 | | APL22 | |  |
| 15 | PRPS2 | 192 | 57 | 14.99 | 1.51% | - |  | 44-57 | | 0.34 | | APL1 | |  |
| 16 | APOA1 | 271 | 0 | 13.73 | 1.41% | - |  | 0-13 | | 0.37 | | APL25 | |  |
| **16** | **ATP5B** | **103** | **2** | **14.57** | **1.01%** | *●* |  | **0-10** | | **0.33** | | - | |  |
| 16 | ENO1 | 105 | 17 | 13.37 | 1.64% | - |  | 9-23 | | 0.35 | | APL22 | |  |
| **16** | PARK7 | **265** | **21** | **14.67** | **0.97%** | *●* |  | **7-23** | | **0.33** | | APL22 | |  |
| **18** | **PGAM1** | **232** | **0** | **17.24** | **1.01%** | *●* |  | **0-12** | | **0.40** | | APL6 | |  |
| 18 | PGAM1 | 325 | 0 | 15.15 | 2.27% | - |  | 0-50 | | 0.35 | | APL6 | |  |
| **18** | **PRDX3** | **257** | **4** | **19.41** | **0.43%** | *●●* |  | **0-12** | | **0.53** | | APL6 | |  |
| 18 | MDH1 | 179 | 11 | 16.42 | 1.40% | - |  | 0-15 | | 0.86 | | APL3 | |  |
| **18** | **ENO1** | **113** | **13** | **25.04** | **0.04%** | *●●●* | ‡‡ | **10-15** | | **1.50** | | APL22 | |  |
| **18** | **GLUL** | **131** | **13** | **17.10** | **1.07%** | *●* |  | **8-16** | | **1.17** | | - | |  |
| 18 | VCP | 285 | 17 | 13.33 | 4.52% | - |  | 0-53 | | 0.63 | | APLZ | |  |
| 18 | GDI2 | 301 | 18 | 16.52 | 1.34% | - |  | 15-29 | | 1.00 | | APL1 | |  |
| **18** | **RPS12** | **321** | **76** | **17.41** | **0.94%** | *●* |  | **63-88** | | **0.45** | | APL3 | |  |
| 18 | PSMB | 215 | 82 | 13.01 | 5.00% | - |  | 73-90 | | 0.39 | | - | |  |
| 18 | SUCLG2 | 127 | 83 | 15.90 | 1.70% | - |  | 74-90 | | 0.44 | | APL13 | |  |
| **18** | **ENO1** | **108** | **85** | **18.73** | **0.56%** | *●* |  | **74-90** | | **0.46** | | APL22 | |  |
| 18 | ENO1 | 106 | 90 | 13.52 | 4.22% | - |  | 82-90 | | 0.35 | | APL22 | |  |
| 18 | EEF2 | 198 | 90 | 13.19 | 4.76% | - |  | 77-90 | | 0.25 | | APL29 | |  |
| 18 | TPI1 | 242 | 90 | 13.52 | 4.22% | - |  | 83-90 | | 0.34 | | APL1 | |  |
| 19 | ACP1 | 274 | 3 | 13.47 | 2.00% | - |  | 0-7 | | 0.34 | | GA3 | |  |
| 19 | EIF3I | 180 | 4 | 13.53 | 1.95% | - |  | 0-11 | | 0.34 | | APL24 | |  |
| 19 | PARK7  1 | 263 | 6 | 12.96 | 2.45% | - |  | 0-41 | | 0.30 | | APL22 | |  |
| 19 | EEF2 | 306 | 19 | 11.25 | 4.69% | - |  | 0-21 | | 1.01 | | APL29 | |  |
| 19 | ME1 | 293 | 20 | 12.16 | 3.33% | - |  | 17-22 | | 1.53 | | APL3 | |  |
| 19 | PRDX6 | 231 | 24 | 11.80 | 3.81% | - |  | 21-41 | | 0.73 | | APL8 | |  |
| 19 | GDI2 | 301 | 41 | 12.40 | 3.04% | - |  | 27-41 | | 0.26 | | APL1 | |  |
| 19 | TBCA | 312 | 41 | 11.88 | 3.70% | - |  | 32-41 | | 0.33 | | APLZ | |  |
| 20 | VCP | 285 | 0 | 11.68 | 4.93% | - |  | 0-9 | | 0.35 | | APLZ | |  |
| **20** | GDI2 | **301** | **18** | **16.37** | **0.80%** | *●* |  | **4-29** | | **0.45** | | APL1 | |  |
| 20 | ACTG1 | 211 | 21 | 14.23 | 1.81% | - |  | 8-43 | | 0.37 | | APL10 | |  |
| 20 | APOA1 | 174 | 31 | 13.80 | 2.17% | - |  | 17-45 | | 0.39 | | APL25 | |  |
| 21 | PARK7 | 263 | 2 | 14.83 | 1.52% | - |  | 0-6 | | 0.44 | | APL22 | |  |
| 21 | RPS12 | 321 | 5 | 13.13 | 2.94% | - |  | 2-10 | | 0.47 | | APL3 | |  |
| 21 | PGAM1 | 230 | 26 | 12.12 | 4.33% | - |  | 16-38 | | 0.33 | | APL6 | |  |
| **21** | **CA2** | **227** | **40** | **20.33** | **0.15%** | *●●* | ‡ | **38-53** | | **0.39** | | APL2 | |  |
| 21 | ENO1 | 112 | 44 | 11.69 | 5.00% | - |  | 22-55 | | 0.35 | | APL22 | |  |
| 21 | HAAO | 196 | 44 | 14.00 | 2.12% | - |  | 40-54 | | 0.41 | | APL3 | |  |
| 21 | ALB | 290 | 55 | 11.94 | 4.63% | - |  | 41-58 | | 0.42 | | APL4 | |  |
| 22 | BPNT1 | 156 | 0 | 11.49 | 2.67% | - |  | 0-10 | | 0.36 | | APL3 | |  |
| **22** | **PRDX4** | **309** | **0** | **14.35** | **0.83%** | *●* |  | **0-3** | | **0.33** | | APL1 | |  |
| 22 | CA2 | 227 | 5 | 10.98 | 3.26% | - |  | 0-10 | | 0.30 | | APL2 | |  |
| **23** | **PDIA3** | **297** | **0** | **18.23** | **0.07%** | *●●●* |  | **0-1** | | **0.33** | | APL10 | |  |
| **23** | **FABP7** | **318** | **0** | **17.35** | **0.09%** | *●●●* |  | **0-1** | | **0.38** | | APL3 | |  |
| 23 | PSMC3 | 116 | 1 | 8.63 | 4.30% | - |  | 0-1 | | 0.31 | | APL5 | |  |
| 23 | NDUFS3 | 228 | 1 | 8.53 | 4.49% | - |  | 0-1 | | 0.30 | | APL5 | |  |
| 23 | APOA1 | 271 | 1 | 11.27 | 1.37% | - |  | 0-1 | | 0.35 | | APL25 | |  |
| 24 | MYH3 | 177 | 0 | 14.66 | 1.12% | - |  | 0-17 | | 0.33 | | - | |  |
| 24 | ACTG1 | 211 | 4 | 12.05 | 3.11% | - |  | 0-16 | | 0.42 | | APL10 | |  |
| **24** | **PGAM1** | **232** | **6** | **15.69** | **0.75%** | *●* |  | **0-16** | | **0.50** | | APL6 | |  |
| **24** | **APOA1** | **174** | **14** | **15.58** | **0.78%** | *●* |  | **6-29** | | **0.36** | | APL25 | |  |
| 24 | PRPS2 | 192 | 15 | 12.67 | 2.44% | - |  | 6-26 | | 0.36 | | APL1 | |  |
| 24 | PSMB | 215 | 18 | 11.20 | 4.30% | - |  | 7-29 | | 0.39 | | - | |  |
| 24 | MDH1 | 179 | 22 | 12.71 | 2.40% | - |  | 8-29 | | 0.39 | | APL3 | |  |
| 24 | ANXA5 | 201 | 22 | 10.98 | 4.67% | - |  | 0-29 | | 0.41 | | APL4 | |  |
| 24 | APOA1 | 262 | 23 | 13.24 | 1.95% | - |  | 6-29 | | 0.37 | | APL25 | |  |
| 24 | PDHB | 185 | 26 | 12.20 | 2.93% | - |  | 10-29 | | 0.42 | | - | |  |
| **24** | **FASN** | **206** | **27** | **29.39** | **0.0018%** | *●●●* | ‡‡ | **19-29** | | **0.68** | | APL19 | |  |
| 24 | ENO1 | 119 | 28 | 11.43 | 3.92% | - |  | 22-29 | | 0.38 | | APL22 | |  |
| 25 | HMGCS2 | 302 | 4 | 11.57 | 4.22% | - |  | 0-30 | | 0.32 | | APLZ | |  |
| 25 | ADK | 136 | 12 | 12.68 | 2.73% | - |  | 0-21 | | 0.35 | | APL6 | |  |
| **25** | **ACADS** | **147** | **17** | **14.97** | **1.09%** | *●* |  | **6-32** | | **0.41** | | APL16 | |  |
| 25 | ANXA2 | 171 | 26 | 10.97 | 5.00% | - |  | 3-39 | | 0.31 | | APL10 | |  |
| **25** | **PARK7** | **269** | **32** | **17.74** | **0.35%** | *●●* |  | **21-39** | | **0.48** | | APL22 | |  |
| 25 | ENO1 | 124 | 34 | 12.19 | 3.32% | - |  | 23-39 | | 0.36 | | APL22 | |  |
| 25 | PRDX4 | 309 | 34 | 11.05 | 5.00% | - |  | 0-39 | | 0.36 | | APL1 | |  |
| 25 | ENO1 | 108 | 38 | 13.11 | 2.29% | - |  | 24-39 | | 0.38 | | APL22 | |  |
| 25 | PRDX4 | 237 | 38 | 12.73 | 2.68% | - |  | 22-39 | | 0.40 | | APL1 | |  |
| **25** | **ENO1** | **304** | **38** | **17.42** | **0.40%** | *●●* |  | **23-39** | | **0.48** | | APL22 | |  |
| **27** | **C11orf54** | **176** | **9** | **17.57** | **0.48%** | *●●* |  | **0-23** | | **0.46** | | APL1 | |  |
| **27** | **MDH1** | **169** | **42** | **17.95** | **0.41%** | *●●* |  | **33-49** | | **0.44** | | APL3 | |  |
| 27 | PSMA2 | 253 | 43 | 11.99 | 4.54% | - |  | 32-51 | | 0.35 | | APL2 | |  |
| 27 | TPI1 | 242 | 51 | 12.51 | 3.70% | - |  | 34-51 | | 0.38 | | APL1 | |  |
| **27** | **TTR** | **324** | **51** | **19.89** | **0.18%** | *●●* |  | **38-51** | | **0.51** | | APL2 | |  |
| 28 | PSMC3 | 116 | 0 | 9.14 | 3.91% | - |  | 0-1 | | 0.35 | | APL5 | |  |
| 28 | ACADS | 147 | 1 | 8.52 | 4.98% | - |  | 0-1 | | 0.37 | | APL16 | |  |
| 28 | EIF3I | 180 | 1 | 9.28 | 3.69% | - |  | 0-1 | | 0.32 | | APL24 | |  |
| 28 | PRDX3 | 257 | 1 | 9.09 | 3.99% | - |  | 0-1 | | 0.36 | | APL6 | |  |
| 28 | GDI2 | 301 | 1 | 10.07 | 1.98% | - |  | 0-1 | | 0.40 | | APL1 | |  |
| 29 | HAAO | 193 | 0 | 10.26 | 4.05% | - |  | 0-10 | | 0.39 | | APL3 | |  |
| 29 | ENO1 | 124 | 7 | 10.33 | 3.94% | - |  | 0-19 | | 0.28 | | APL22 | |  |
| 29 | PSMB | 215 | 8 | 10.71 | 3.40% | - |  | 0-19 | | 0.32 | | - | |  |
| **29** | **VDAC1** | **216** | **8** | **19.06** | **0.09%** | *●●●* |  | **0-13** | | **0.46** | | - | |  |
| 29 | **PARK7** | 265 | 8 | 9.75 | 4.97% | - |  | 0-19 | | 0.32 | | APL22 | |  |
| 29 | SOD2 | 267 | 9 | 12.85 | 1.43% | - |  | 0-17 | | 0.40 | | - | |  |
| 29 | TPI1 | 242 | 13 | 11.38 | 2.58% | - |  | 0-19 | | 0.34 | | APL1 | |  |
| 29 | GLUL | 131 | 13 | 10.16 | 4.23% | - |  | 7-18 | | 0.29 | | - | |  |
| **29** | **GLUL** | **128** | **14** | **14.85** | **0.64%** | *●* |  | **8-18** | | **0.36** | | - | |  |
| 29 | PRDX6 | 231 | 18 | 10.33 | 3.94% | - |  | 0-19 | | 0.34 | | APL8 | |  |
| Z | GLUD1 | 319 | 6 | 15.65 | 1.13% | - |  | 0-12 | | 0.59 | | APL6 | |  |
| Z | ANXA5 | 201 | 20 | 16.28 | 0.88% | - |  | 17-26 | | 0.38 | | APL4 | |  |
| Z | PARK7 | 263 | 20 | 13.13 | 3.01% | - |  | 18-32 | | 0.38 | | APL22 | |  |
| Z | ENO1 | 144 | 22 | 11.90 | 4.84% | - |  | 13-35 | | 0.34 | | APL22 | |  |
| **Z** | **TPI1** | **236** | **22** | **17.16** | **0.63%** | *●* |  | **18-35** | | **0.45** | | APL1 | |  |
| Z | ALDH7A1 | 300 | 30 | 11.80 | 4.99% | - |  | 22-35 | | 0.31 | | APLZ | |  |
| Z | ATP5B | 103 | 32 | 13.61 | 2.51% | - |  | 31-35 | | 0.36 | | - | |  |
| Z | PSMA1 | 212 | 32 | 14.12 | 1.14% | - |  | 31-35 | | 0.43 | | APL5 | |  |

^1^ Duck (Anas Platyrhynchos) chromosome or linkage group.

^2^ Protein descriptions: see supplementary data

^3^ Spot number on the 2D gels.

^4^ Position on the genetic map in centiMorgans.

^5^ Level of significance of P-value: chromosome-wide: ● 0.01 > P > 0.005; ●● 0.005 > P > 0.001; ●●● 0.001 > P and genome-wide: ‡0.05 > P > 0.01; ‡‡ 0.01 > P.

^6^ Position of the corresponding gene in Anas Platyrhyncos; underlined if pQTL and gene in the same APL chromosome.
