# Supplementary material for: Deciphering mechanisms underlying the genetic variation of general production and liver quality traits in the overfed mule duck by pQTL analyses
Source: Genet Sel Evol. 2017 Apr 19;49:38. doi: 10.1186/s12711-017-0313-6 (PMC5396126; doi:10.1186/s12711-017-0313-6)
Supplement: Supplementary file 3 — Additional file 3: Table S3. Single-trait QTL detection of zootechnical traits. List of 80 QTL with APL chromosome number, protein name, spot number, QTL location (cM), maximum likelihood ratio, P-value, threshold reached, confidence interval, substitution effect and gene position on APL chromosome. [file 12711_2017_313_MOESM3_ESM.docx]

**Table S3: single-trait QTL detection of zootechnical traits**

| **APL^1^** | **Trait** | **Location**  **(cM)^2^** | **LRTx** | ***P*-value** | **Threshold^3^** | | **Confidence interval** | **Subst. Effect** |
| --- | --- | --- | --- | --- | --- | --- | --- | --- |
| 1 | GLU 10^th^ M | 64 | 16.79 | 3.41% | - |  | 33-65 | 0.59 |
| 1 | CHO 10^th^ M | 83 | 16.17 | 4.31% | - |  | 72-87 | 0.66 |
| **1** | **BW12** | **106** | **20.12** | **0.90%** | *●* |  | **99-119** | **0.46** |
| 1 | BW28 | 107 | 17.53 | 2.61% | - |  | 99-119 | 0.42 |
| 2 | La* | 20 | 16.70 | 2.68% | - |  | 10-32 | 0.44 |
| **2** | **MR** | **63** | **27.75** | **0.03%** | *●●●* | ‡‡ | **58-67** | **0.60** |
| **2** | **LProtC** | **64** | **21.59** | **0.37%** | *●●* | ‡ | **58-67** | **0.50** |
| **2** | **FLW** | **64** | **21.91** | **0.34%** | *●●* | ‡ | **58-68** | **0.54** |
| **3** | **BWG12-42** | **44** | **20.93** | **0.64%** | *●* |  | **23-53** | **0.47** |
| **3** | **BWG12-28** | **44** | **21.44** | **0.53%** | *●●* |  | **18-68** | **0.49** |
| 3 | BW70 | 44 | 16.63 | 3.58% | - |  | 15-53 | 0.34 |
| **3** | **BW28** | **44** | **22.18** | **0.38%** | *●●* | ‡ | **23-68** | **0.50** |
| **3** | **BW42** | **44** | **23.78** | **0.20%** | *●●* | ‡ | **26-54** | **0.51** |
| 3 | BW12 | 61 | 16.73 | 3.44% | - |  | 49-70 | 0.60 |
| 3 | TG 20^th^ M | 63 | 17.95 | 2.19% | - |  | 61-70 | 0.50 |
| 3 | LLipC | 117 | 17.76 | 2.36% | - |  | 112-119 | 0.49 |
| **3** | **LProtC** | **117** | **20.24** | **0.85%** | *●* |  | **112-121** | **0.45** |
| 5 | BWG12-42 | 0 | 9.72 | 4.18% | - |  | 0-2 | 0.27 |
| 5 | TG 2^nd^M | 0 | 12.66 | 1.25% | - |  | 0-2 | 0.38 |
| 5 | AFW | 1 | 9.42 | 4.84% | - |  | 0-2 | 0.34 |
| 6 | MLipC | 0 | 15.25 | 1.67% | - |  | 0-8 | 0.39 |
| 6 | LColC | 8 | 14.61 | 2.18% | - |  | 0-19 | 0.39 |
| 6 | LL* | 14 | 14.22 | 2.54% | - |  | 3-30 | 0.40 |
| 7 | GLU 10^th^ M | 14 | 15.01 | 2.63% | - |  | 0-32 | 0.40 |
| 7 | CHO 20^th^ M | 30 | 14.30 | 3.43% | - |  | 27-38 | 0.71 |
| **7** | **TSW** | **40** | **18.21** | **0.80%** | *●* |  | **5-70** | **0.41** |
| 7 | BWbeg | 60 | 13.57 | 4.54% | - |  | 32-70 | 0.40 |
| 8 | Menergy | 6 | 14.95 | 2.72% | - |  | 0-11 | 0.60 |
| 8 | CHO 20^th^ M | 63 | 14.59 | 3.11% | - |  | 60-64 | 0.42 |
| 9 | LLipC | 38 | 13.43 | 2.72% | - |  | 33-45 | 0.32 |
| **9** | **OWG** | **40** | **23.44** | **0.05%** | *●●●* | ‡ | **38-47** | **0.53** |
| 9 | pmSFW | 40 | 12.44 | 4.06% | - |  | 36-47 | 0.31 |
| 9 | MCookL | 43 | 14.44 | 1.79% | - |  | 38-47 | 0.39 |
| 9 | FLW | 45 | 12.30 | 4.29% | - |  | 33-47 | 0.33 |
| 9 | BW12 | 47 | 13.70 | 2.45% | - |  | 38-47 | 0.34 |
| **10** | **GLU 2^nd^ M** | **40** | **16.17** | **0.81%** | *●* |  | **37-40** | **0.37** |
| 11 | pmMW | 16 | 13.72 | 2.99% | - |  | 11-29 | 0.55 |
| 12 | CHO 2^nd^ M | 98 | 13.57 | 3.33% | - |  | 89-128 | 0.41 |
| 12 | pmSFW | 100 | 12.84 | 4.37% | - |  | 89-108 | 0.39 |
| 12 | MLipC | 137 | 13.61 | 3.28% | - |  | 130-143 | 0.28 |
| 12 | Mb* | 138 | 15.36 | 1.70% | - |  | 126-147 | 0.26 |
| 13 | Ma* | 60 | 13.56 | 1.66% | - |  | 55-72 | 0.34 |
| 14 | CW | 0 | 11.68 | 4.81% | - |  | 0-10 | 0.38 |
| 14 | Mb* | 0 | 12.38 | 3.67% | - |  | 0-18 | 0.39 |
| 14 | MLipC | 5 | 13.35 | 2.53% | - |  | 0-27 | 0.43 |
| 14 | BWG42-70 | 16 | 12.26 | 3.84% | - |  | 3-26 | 0.32 |
| 14 | TG 2^nd^ M | 31 | 13.44 | 2.44% | - |  | 27-47 | 0.30 |
| 14 | CHO 20^th^ M | 69 | 11.62 | 4.90% | - |  | 44-69 | 0.37 |
| 15 | CHO 2^nd^ M | 39 | 12.41 | 4.94% | - |  | 23-54 | 0.35 |
| 15 | Lb* | 38 | 15.31 | 1.66% | - |  | 27-57 | 0.41 |
| 15 | LProtC | 39 | 14.18 | 2.58% | - |  | 29-57 | 0.42 |
| **15** | **MR** | **41** | **17.10** | **0.77%** | *●* |  | **31-57** | **0.45** |
| 15 | LLipC | 42 | 15.70 | 1.39% | - |  | 30-56 | 0.44 |
| 16 | FLW | 0 | 12.11 | 2.77% | - |  | 0-14 | 0.34 |
| 16 | LColC | 1 | 12.00 | 2.90% | - |  | 0-4 | 0.33 |
| 18 | MvacL | 16 | 13.39 | 4.26% | - |  | 13-23 | 1.04 |
| 18 | CortL | 20 | 13.05 | 4.86% | - |  | 0-40 | 0.51 |
| **18** | **Lb*** | **67** | **24.07** | **0.06%** | *●●●* | ‡ | **42-69** | **0.39** |
| 19 | LProtC | 0 | 10.61 | 4.37% | - |  | 0-7 | 0.33 |
| 19 | BWG12-28 | 4 | 11.21 | 3.39% | - |  | 0-7 | 0.31 |
| 19 | CHO 2^nd^ M | 4 | 11.29 | 3.31% | - |  | 0-21 | 0.31 |
| 19 | BW28 | 4 | 11.83 | 2.71% | - |  | 0-8 | 0.34 |
| 21 | BWend | 6 | 12.28 | 4.08% | - |  | 4-15 | 0.37 |
| 21 | MFmax | 53 | 11.87 | 4.84% | - |  | 18-58 | 0.40 |
| 22 | BWG28-42 | 9 | 10.61 | 3.87% | - |  | 0-10 | 0.32 |
| **22** | **Lb*** | **9** | **14.27** | **0.98%** | *●* |  | **0-10** | **0.32** |
| **23** | **LLipC** | **0** | **14.59** | **0.30%** | *●●* |  | **0-2** | **0.35** |
| **23** | **LProtC** | **0** | **15.46** | **0.19%** | *●●* |  | **0-2** | **0.38** |
| 23 | MR | 0 | 11.72 | 1.11% | - |  | 0-2 | 0.32 |
| **23** | **TG 2^nd^ M** | **1** | **14.39** | **0.32%** | *●●* |  | **0-2** | **0.34** |
| 23 | FLW | 1 | 9.73 | 2.63% | - |  | 0-2 | 0.30 |
| 24 | GLU 20^th^ M | 11 | 13.41 | 2.92% | - |  | 0-22 | 0.45 |
| 24 | CHO 2^nd^ M | 28 | 15.00 | 1.81% | - |  | 20-29 | 0.39 |
| **25** | **La*** | **6** | **15.61** | **0.92%** | *●* |  | **0-17** | **0.35** |
| **27** | **MvacL** | **34** | **18.64** | **0.36%** | *●●* |  | **30-42** | **0.44** |
| 27 | GLU 10^th^ M | 50 | 13.28 | 3.33% | - |  | 48-51 | 0.37 |
| Z | GLU 2^nd^ M | 1 | 15.79 | 1.22% | - |  | 0-5 | 0.43 |
| Z | CHO 10^th^ M | 13 | 13.17 | 3.39% | - |  | 6-20 | 0.35 |
| Z | GLU 10^th^ M | 27 | 13.14 | 3.42% | - |  | 24-35 | 0.39 |
| **Z** | **MpHu** | **32** | **19.44** | **0.29%** | *●●* |  | **31-35** | **0.66** |

^1^ Duck (Anas Platyrhynchos) chromosome or linkage group

^2^ Position on the genetic map in centiMorgans.

^3^ Level of significance of P-value: chromosome-wide: ● 0.01 > P > 0.005; ●● 0.005 > P > 0.001; ●●● 0.001 > P and genome-wide: ‡0.05 > P > 0.01; ‡‡ 0.01 > P.
